# Active thermo-reflectometry for absolute temperature measurement by infrared thermography on specular materials

**DOI:** 10.1038/s41598-022-11616-8

**Published:** 2022-05-12

**Authors:** Thomas Lafargue-Tallet, Romain Vaucelle, Cyril Caliot, Abderezak Aouali, Emmanuelle Abisset-Chavanne, Alain Sommier, Raymond Peiffer, Christophe Pradere

**Affiliations:** 1 I2M TREFLE, UMR 5295 CNRS-UB-ENSAM, 351 Cours de la Libération, 33400 Talence, France; 2 MBDA France, 1 Avenue Réaumur, 92350 Le Plessis-Robinson, France; 3 EPSILON - Groupe ALCEN, Esplanade des Arts et Metiers , 33405 Talence Cedex, France; 4 Universite de Pau et des Pays de l’Adour, E2S UPPA, CNRS, LMAP, Anglet, France

**Keywords:** Energy science and technology, Engineering, Materials science, Optics and photonics, Applied physics

## Abstract

Knowledge of material emissivity maps and their true temperatures is of great interest for contactless process monitoring and control with infrared cameras when strong heat transfer and temperature change are involved. This approach is always followed by emissivity or reflections issues. In this work, we describe the development of a contactless infrared imaging technique based on the pyro-reflectometry approach and a specular model of the material reflection in order to overcome emissivities and reflections problems. This approach enables in situ and real-time identification of emissivity fields and autocalibration of the radiative intensity leaving the sample by using a black body equivalent ratio. This is done to obtain the absolute temperature field of any specular material using the infrared wavelength. The presented set up works for both camera and pyrometer regardless of the spectral range. The proposed method is evaluated at room temperature with several heterogeneous samples covering a large range of emissivity values. From these emissivity fields, raw and heterogeneous measured radiative fluxes are transformed into complete absolute temperature fields.

## Introduction

**Figure 1:**
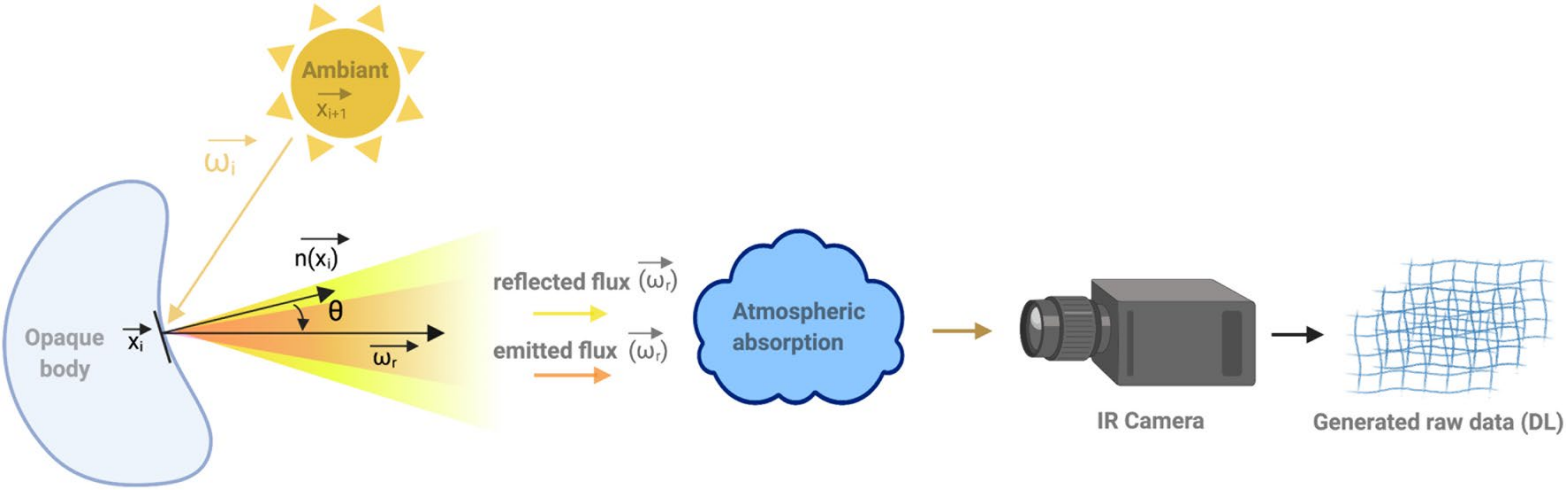
Schema of the main difficulties in the contactless measurement of a thermal scene by using IR thermography.

Knowledge of absolute temperature mapping is a key parameter in many industrial applications, such as additive manufacturing, material shaping, and thermal processes. Very often, the process itself or its environment (extreme conditions, temperature range, cluttering of the environment, etc.) do not allow contact instrumentation using conventional methods such as thermocouples, thermopiles or other intrusive techniques. Thus, for such applications, and more generally in the new con.text of the future 4.0 industry, it is necessary to be able to develop imaging contactless methods adapted to complex shapes and heterogeneous samples. Among the contactless methods in the infrared (IR) spectral range, pyrometry and thermography are the most popular and are based on radiometric methods and models. Then, regardless of the radiometric techniques used, the simple measurement of the flux emitted by a surface or a thermal scene does not easily allow determination of its absolute temperature. Indeed, as depicted in Fig. 1, the surface spectral and directional radiative properties depend on their optical properties (refractive indexes) and surface roughness and their variations due to temperature change. In addition to these properties, the radiative fluxes in a scene depend on the temperature field, the scene geometry and the participating atmosphere (which is considered transparent here). Thus, when considering only opaque surfaces (which is the case in this study), the radiative flux measured by any IR sensor (see Fig. 1) depends on emission, absorption, multiple reflection and multiple scattering. Even if one succeeds in controlling, measuring or determining all of these parameters, it is also important to obtain the calibration curve of the IR sensors, which has a nonlinear dependency on the absolute temperature.

In this context, and with regard to the literature, one can note that today, few experimental techniques allow for a simple, reliable, robust and precise measurement of the absolute temperature by contactless infrared sensors, and more precisely by imaging methods based on IR cameras.

Indeed, pyrometric measurements of radiance to determine temperature have been performed for decades^1^. From the beginning, the characterization of emissivity has always been a major issue^2^. To overcome this issue, a bichromatic^3^ pyrometer has been implemented, which has been extended to a multispectral pyrometer^4,5^. These methods assume that the evolution of the emissivity between nearby wavelengths is constant or known. In addition to radiance measurements, the evaluation of the directional hemispherical reflectivity^6^ was introduced to deduce both emissivity and temperature^7–10^. The adjustment of these methods for matricial sensors is still an ongoing endeavour. To do so, in early 2000, the pyro-reflectometry^9,11^ method was proposed by pioneer and adapted to an infrared camera with the thermo-reflectometry method^12,13^, which requires the measurement of bidirectional reflectivity at two specific wavelengths in the near-infrared (NIR) region and assumes that the bidirectional reflectance distribution function (BRDF) is constant between nearby wavelengths. Others method are based on correlation between visible and infrared observation. This method use teaching learning^14^ algorithm or cross correlation invariant coefficient between both spectrum^15^. Vellvehi et al.^16^ conducted irradiance-based emissivity correction based on two reference thermal images obtained at two uniform temperatures.

In addition to the work carried out on emissivity correction, some methods have been developed to estimate parasitic reflection that occurs during temperature measurement in highly reflective scenes^17,18^ at high temperatures. This method are based on radiation models^19^. Other method had been developed by Vollmer and al.^20^ by studying the polarisation of reflection in order to set up a polariser filter. Other methods have been implemented for infrastructure surveillance (especially tunnels) to eliminate reflections from the environment using a mobile camera and a dedicated algorithm^21^.

If the use and the technology offered by IR cameras are in full development and implementation today. The analysis of the state of the art highlights the great difficulty of obtaining a robust and real-time measurement of the absolute temperature of thermal scenes acquired with IR imagers in a simple way overcoming both reflection and emissivity problems. In this article, a method answering this issue is presented on any specular material in infrared wavelength.

This model, which is widely applicable in the industry in regards of proposed roughness and flatness allows to determine both experimentally emissivity and reflexions without any assumption. The ease of set up realization as well as the ease of implementation (subtraction or division of images) make it an easy and versatile method transposable in industrial process .

In this article, a method of simultaneous measurement of emissivity maps by multispectral thermo-reflectometry and raw radiative intensity by IR thermography is presented. This work is based on the development of a new measurement system based on an active optical approach that consists of illuminating the objects by means of black body beam sources expanded on the surface of the materials with incidence normal to the surface or thermal scene to be measured. This source coupled with methods known as 2 images^22,23^ (by means of a mechanical chopper) will allow the simultaneous measurement of the normal reflectivity of the object to the multispectral source, as well as its own proper emission at the classical frequency of acquisition of current cameras. This approach, coupled with a specific calibration of the environment and associated with a method based on a model of specular reflection of surfaces, will allow for obtaining emissivity fields in real time. Knowledge of these fields associated with the measurement of the radiative intensity and a black body calibration will allow determination of contactless absolute temperature fields.

This work presents (i) a description of the method via a formal development based on radiative transfer and the notion of the bidirectional reflectance distribution function (BRDF) and (ii) a presentation of the samples used, as well as the measurement device. Then, in the second part, the different calibration methods of the camera, the measurements and the environment will be presented, and validation will be presented through an example of a specular homogeneous medium. Finally, in the last part, the application of the method is carried out on two metallic samples with very different surface heterogeneities ranging from black to polished metal.

## Material and method

### Experimental set-up

**Figure 2:**
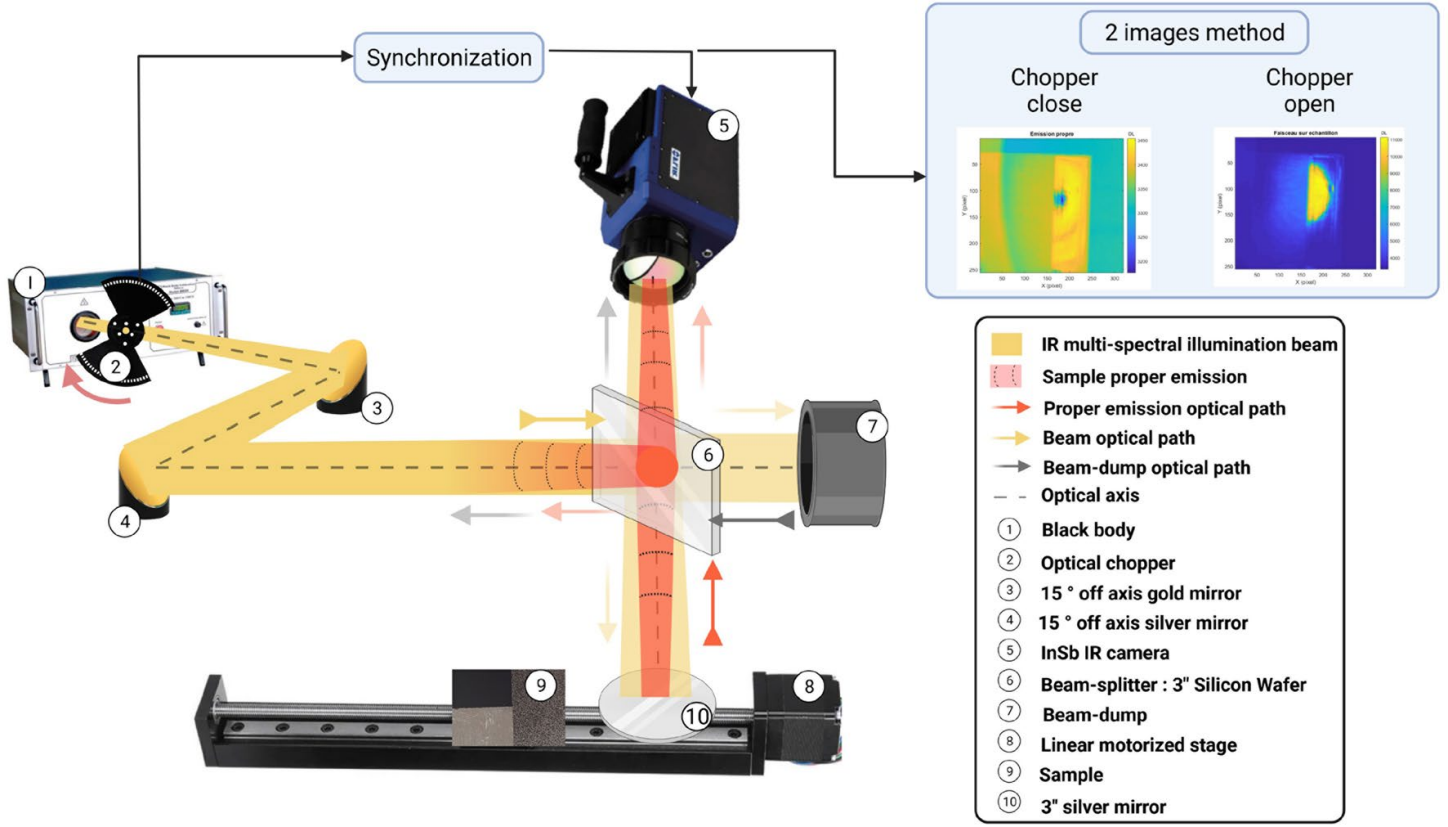
Schematic representation of the experimental setup.

The complete setup used to measure the normal radiative intensity leaving a sample is described in Fig. 2. The experimental bench is composed of (1), a multispectral black body source (1: CN-MT provided by Prisma Instrument) whose temperature range extends from 20 to 500 $^\circ$C. In this study, the temperature set point was 200 $^\circ$C. The first advantage of the black body illumination source is the ability to adjust the illumination’s power according to the signal needed to reach a good measurement in term of signal to noise ratio (few reflectance or high temperature specimen). The second advantages is that this equipment emits overall the IR spectrum and multiple cameras can be used according to the best spectral range in term of materials or sensitivity. A chopper (2) is used to modulate the intensity of the beam in lock-in mode. Typically, the frequency modulation ($f_{BB}$) was set to 50 Hz, whereas the camera frequency acquisition ($f_{acq}$) was set to double this value. A pair of parabolic mirrors (3 and 4) is used as an optical doublet to image a plane of the black body cavity onto the sample plane. The object plane (black body cavity) of the doublet is imaged on the image plane (sample) with a theoretical magnification of 2. As the diameter of the black body cavity is approximately 25.4 mm, the beam that illuminates the sample surface reaches a theoretical diameter of 50 mm. Mirror (3) is a 15$^\circ$ gold-coated off axis with a focal length of 645,92 mm, whereas mirror (4) is a 15$^\circ$ off axis with a focal length of 542,92 mm and has a silver coating. As the optical set up contains only mirror, there is no chromatic aberration. The IR camera (5), model SC7000 (synchronized with the optical chopper: $f_{acq}=2\,f_{BB}$), has an InSb sensor (2,5–5,5 $\mu m$) with a frame resolution of 320 $\times$ 256 pixels and a pitch of 30  $\mu m$ and is equipped with a 50  mm focal lens for a spatial resolution of $300 \mu m$. The beam splitter (6) (with $50\%$ transmissivity, $50\%$ reflectivity) is used to both normally illuminate the sample and to record the normal radiative intensity leaving the sample with the IR camera. A motorized rail (8) allows linear motion of the reference mirror (9) and the sample (10). Both are mounted with particular attention on coplanar alignments that are normal to the camera and beam axis.

Due to the beam splitter, the camera field of view is the superposition of the sample plane (transmitted by the beam splitter) and the beam dump (reflected by the beam splitter) plane. To minimise the beam dump participation, it is positioned far from the camera focal plane and painted with an absorbing black paint to avoid any reflection. The specimen is placed 1300 mm away from the camera.

### Presentation of the samples

**Figure 3:**
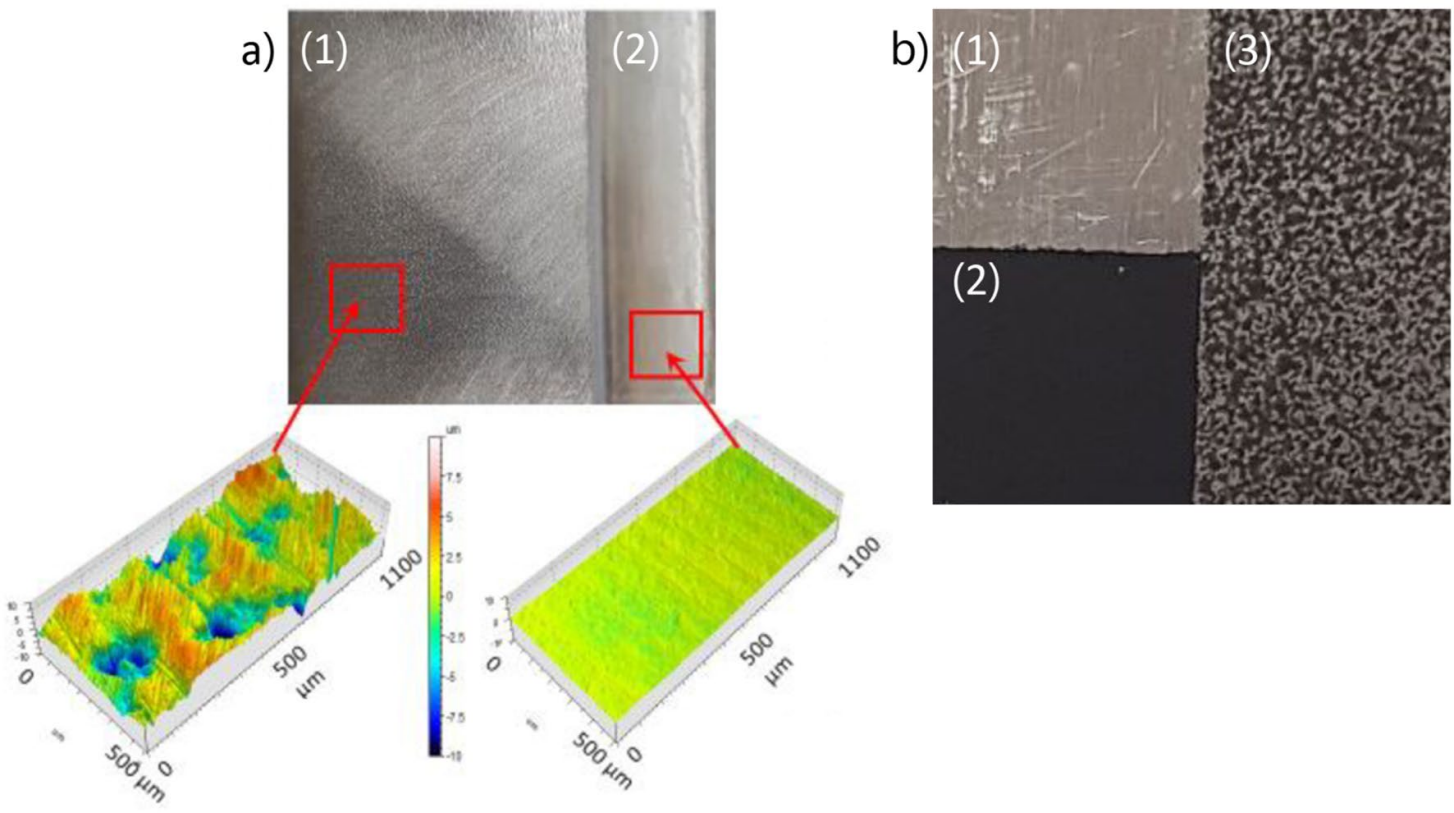
Photography of the sample characterized in this paper: (**a**) two-part stainless steel sample with (1), a textured surface with an apparent roughness of 6 $\mu m$ and (2), polished surface with a roughness of 320 nm^24^ and (**b**), three-part aluminum sample with (1), raw surface, (2), black painted surface with 0.92 emissivity and (3), surface speckled with the same black paint.

As depicted in Fig. 3, two different opaque metallic samples were used in this study. The first picture in Fig. 3.a represents the first sample, which is a stainless steel cubic sample with two different surface treatments: (i) a rough part (Fig. 3.a.1) and (ii) a laser polished surface (Fig. 3.a.2). A detailed description of the samples is given in^24^. The second sample (see Fig. 3.b) is homemade heterogeneous aluminum divided into three parts: (i) is a raw surface (Fig. 3.b.1); (ii) is uniformly painted in black (Fig. 3.b.2); and (iii) is speckled with the same paint (Fig. 3.b.3). The paint used is a matte black (RAL 9005) whose emissivity was estimated to be approximately 0.92, as depicted in reference^25^. The size of both samples is approximately 60 mm square.

### Black body calibration

Whatever the kind of infrared sensor we used, the raw data measured are provided in Digital Level (DL). This arbitrary unit is proportional to the radiative intensity ($I_b$: $W.m^{-2}.sr^{-1}.\mu m ^{-1}$) of the black body under certain conditions of temperature ($T_b$: *K*), integration time (IT, $t_{IT}$: $\mu s$), optics, etc. $I_b$ and $T_b$ are both linked by Planck’s law described in Eq. 1:

1 $$\begin{aligned} I_b(\lambda ,T)=\frac{2\,h\,c^2}{\lambda ^5} \frac{1}{e^{\frac{h\,c}{\lambda T k_B }}-1} \end{aligned}$$

Where *h* is the Planck constant ($m^2.kg.s^{-1}$), $k_B$ is the Boltzmann constant ($W.m^{-2}.K^{-4}$), *c* is the speed of light ($m.s^{-1}$) in air and $\lambda$ is the wavelength ($\mu m$).

**Figure 4:**
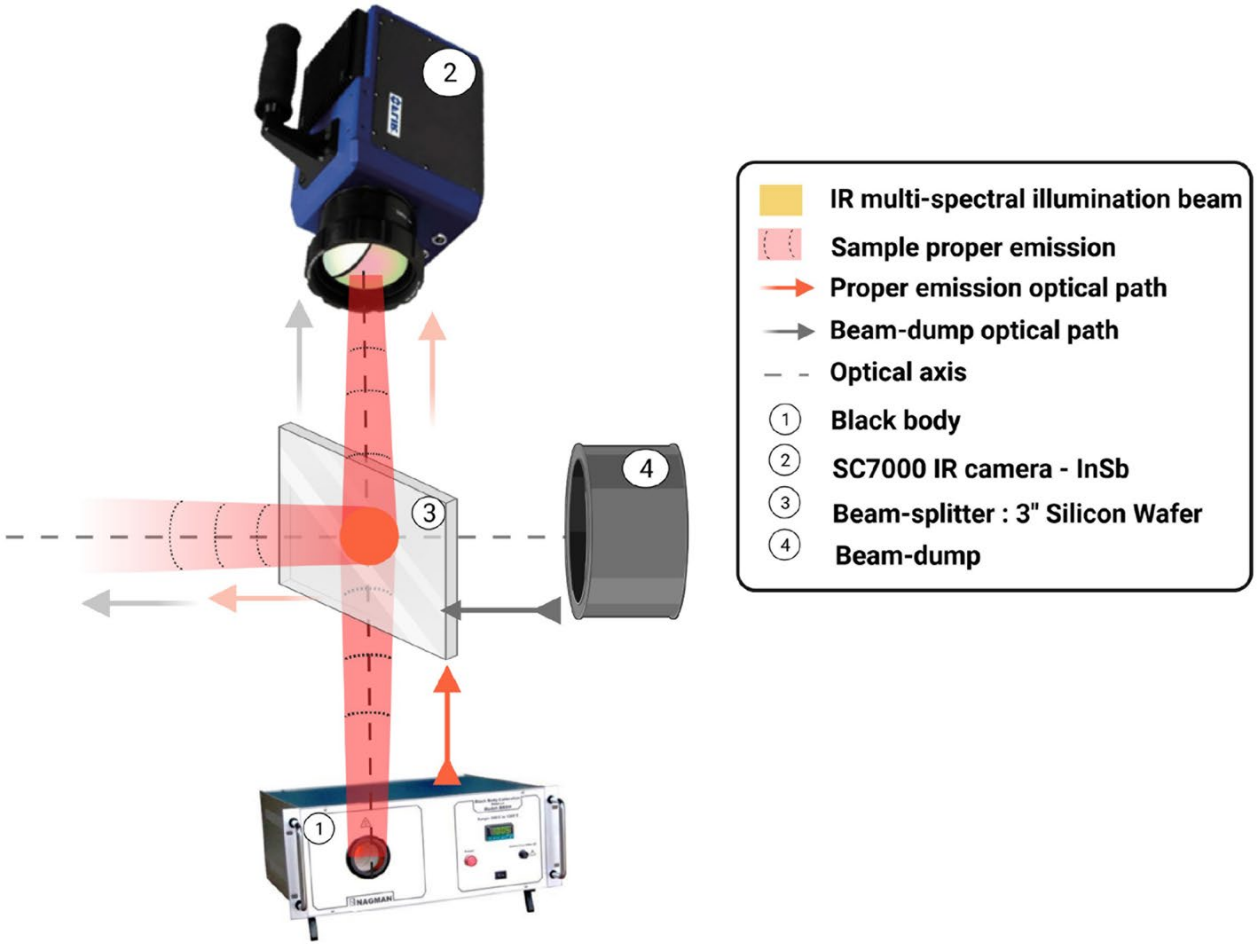
Experimental setup during calibration procedure.

Thus, even without making assumptions regarding the surface conditions and/or other problems of sample emissivity, the first step is the calibration of the camera to determine the relation between the measured DL and the true temperature of a black body. This step can be performed by the camera supplier, but in our case, the option of self-calibration of the entire measurement chain has been retained (see Fig. 4). The literature regarding IR camera calibration is abundant^26–32^. There are multiple parameters influencing the calibration, such as the object-to-camera distance^33^, the object orientation^34^, the detector type^26,35^ and the magnification^36^.

As described in Fig. 4, the experimental setup used for the calibration phase is a part of the complete setup shown in Fig. 2. This method allows us to integrate all the reflections, especially the absorption of the radiative intensity emitted by the objects through the separating plate.

Indeed, the calibration setup takes into account the transmissivity of the beam splitter ($\tau _{bs}$) and the background reflected by the beam splitter ($I_{bs}$ is considered negligible because it is far from the sharp field of view of the camera).

The camera transfer function (*f*) is unknown (described in Eq. 2) and dependent on $\tau _{bs} I_b$ and the black body temperature *T*. One must find a direct link between *T* and DL through the calibration (*g*) described in Eq. 4.

2 $$\begin{aligned} DL= & {} f\big (\tau _{bs}\,I_b(T,\lambda )\big ) \\ \end{aligned}$$

3 $$\begin{aligned} T= & {} g(DL) \end{aligned}$$

**Figure 5:**
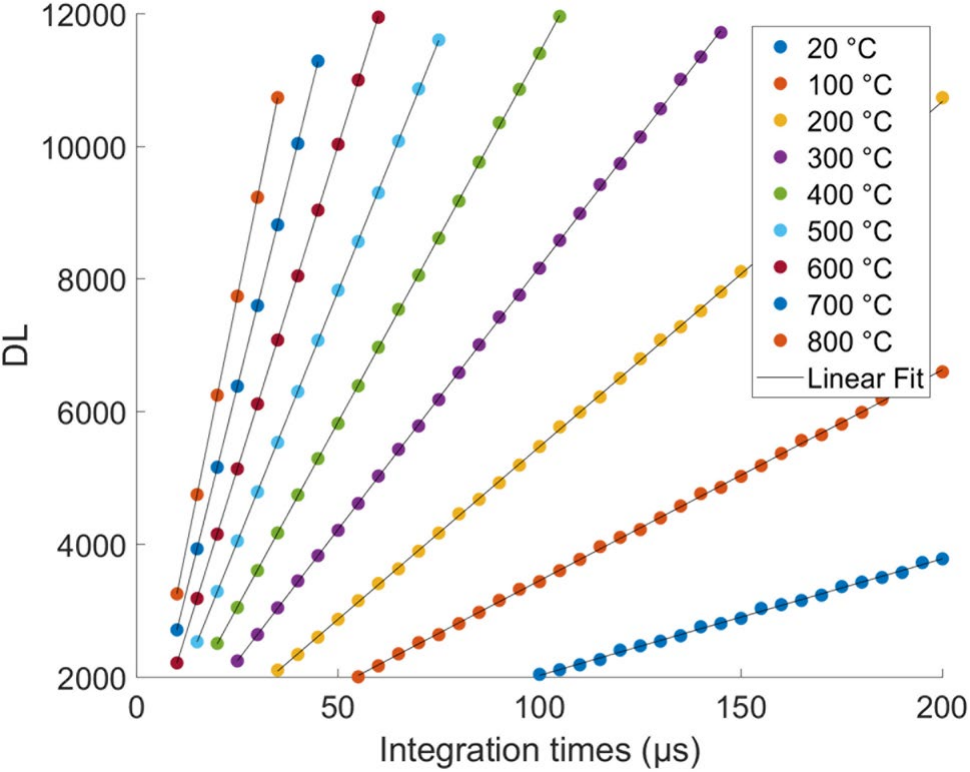
Set of curves obtained during black body calibration.

The calibration procedure includes integration times varying from 10 to $200~\mu s$ and temperatures from 20 to 800 $^\circ$C. Then, a set of curves is obtained describing the temperature in terms of DL for multiple integration times, as shown in Fig. 5.

As one can observe in Fig. 5, the camera response seems to be a linear function of the integration time with temperature dependencies of the slope coefficient and the origin ordinate, as written in Eq. 5.

4 $$\begin{aligned} f=a(T)\,t_{IT}+b(T) \end{aligned}$$

**Figure 6:**
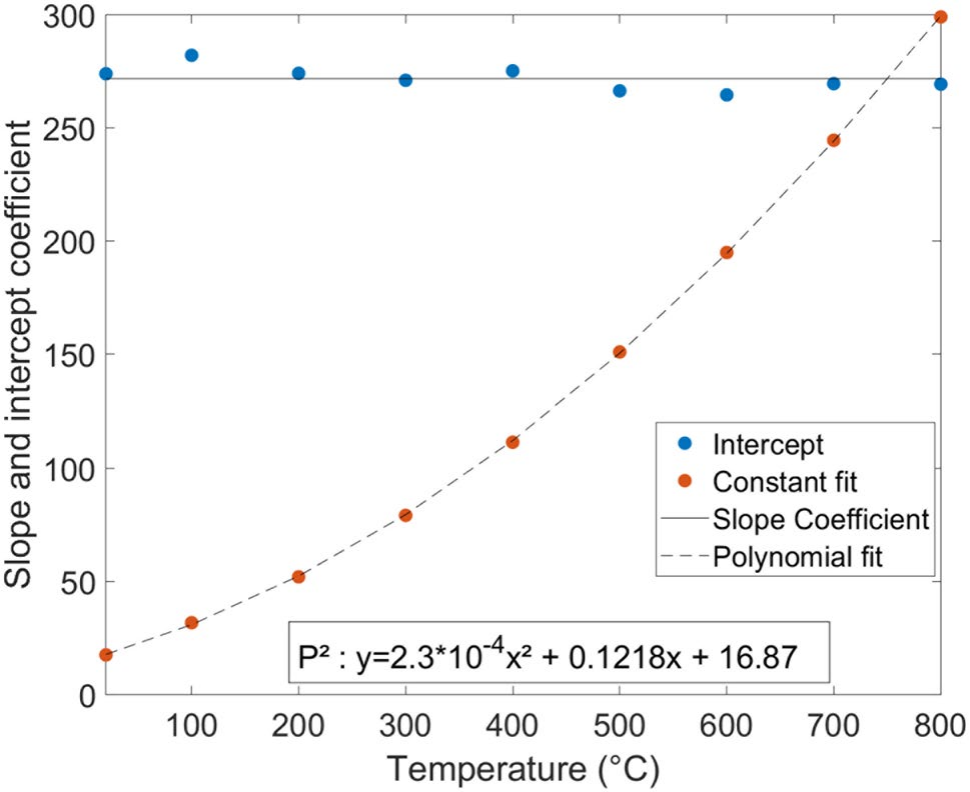
Temperature evolution of the coefficient of the linear regression of Eq. 5.

The temperature evolution of the coefficient of the linear regression of Eq. 5 is reported for the slope and the origin ordinate in Fig. 6.

The origin ordinate is considered constant and averaged. The slope coefficient is fitted with ($p^2$), a polynomial interpolation of order 2. Thus, Eq. 5 can be written as (see Eq. 6).

5 $$\begin{aligned} f=p^2(T)\,t_{IT}+272 \end{aligned}$$

Once this transfer function is obtained, we must identify the temperature as a function of DL (*g*). To do so, thanks to Eq. 6, one can create an abacus of curves for each IT that one has not measured. For each one, a fit is determined and stored as calibration (*g*).

**Figure 7:**
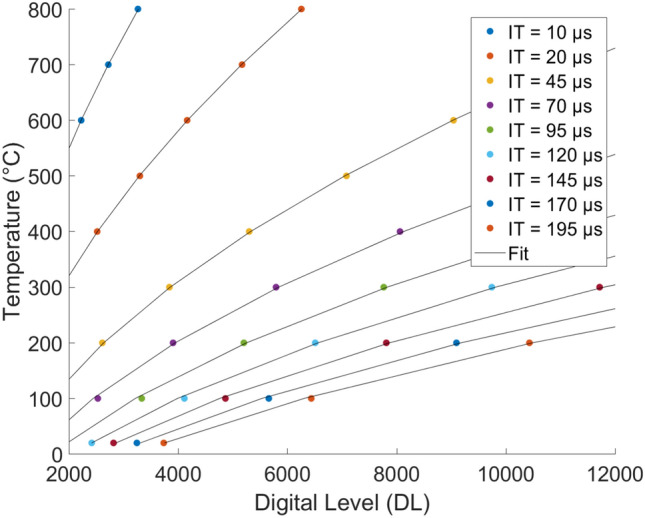
Complete calibration curve linking the DL measured by the IR camera with absolute black body temperature as a function of the IT.

To finish, in order to validate the calibration, one can project the obtained fit on different known experimental points, as shown in Fig. 7. In this case, the relative mean deviation is approximately $0.8~\%$.

## Radiative model

### Description of the model

**Figure 8:**
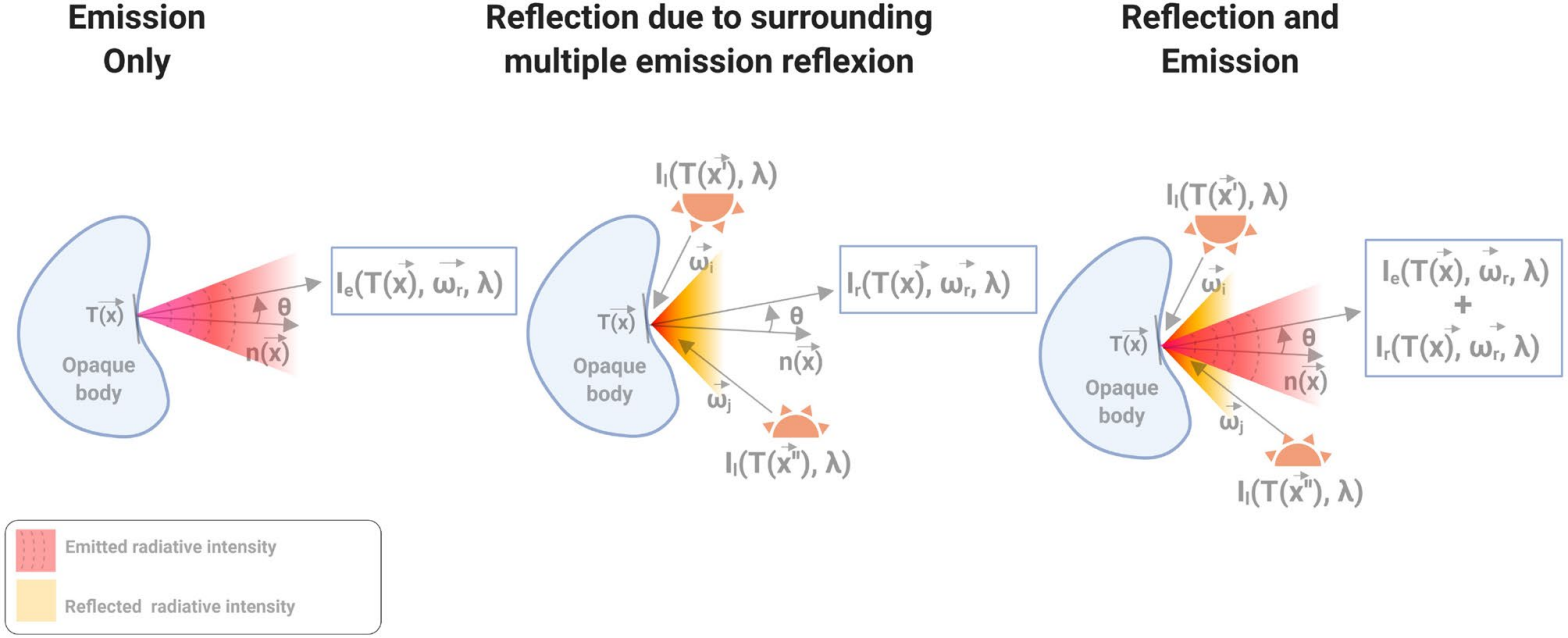
Schematic of the radiative problem of an opaque body at ambient temperature placed into an environment with notations used for ray reflection on an elementary surface at $\vec {x}_{i}$ (incident direction $\vec {\omega }_i$, reflected direction $\vec {\omega }_r$).

The problem of contactless measurement of absolute temperature by an IR camera is a rather complex problem that we illustrate by the diagram in Fig. 8. Indeed, the raw data expressed in digital levels (DL) measured by an IR camera are derived from the incident radiation on the camera detector, which comes from emission, transmission and multiple reflections occurring inside the studied scene (Fig. 8). These DL are then converted to temperature using calibration curves identified with a black body (see Sect. 2.3).

Considering a **transparent** atmosphere and **opaque** surfaces, the radiative intensity leaving a surface ($I_{l}$: $W.m^{-2}.sr^{-1}.\mu m^{-1}$) at $\vec {x}$ (with outward normal $\vec {n}$) and incident to the camera in direction $\vec {\omega }_r$ (Fig. 8) is the sum of emitted ($I_e$: $W.m^{-2}.sr^{-1}.\mu m^{-1}$) and reflected ($I_{r}$: $W.m^{-2}.sr^{-1}.\mu m^{-1}$) radiative intensities (see Eq. 7). Both quantities depend on the local temperature since emissivity, reflectivity or Planck’s law depend on it.

6 $$\begin{aligned} I_l(T(\vec {x}),\vec {\omega }_{r},\lambda )= & {} I_e\big (T(\vec {x}),\vec {\omega }_{r},\lambda \big ) + I_{r}\big (T(\vec {x}),\vec {\omega }_{r},\lambda \big ) \end{aligned}$$

Due to the beam splitter (Fig. 2) in the optical path from the sample to the IR camera, the monochromatic radiative intensity arriving at the camera ($I_l$) results in the superposition of the radiative intensity leaving the sample surface at $\vec {x}$ towards the camera (Eq. 7, which is transmitted by the beam splitter, $\tau _{bs}$) and the background radiative intensity reflected by the beam splitter. This background intensity, $I_{bd}$, comes from the beam dump emission and reflections.

7 $$\begin{aligned} I_l\big (T(\vec {x}),\vec {\omega }_{r},\lambda \big ) = \tau _{bs} \bigg [ I_e\big (T(\vec {x}),\vec {\omega }_{r},\lambda \big ) + I_{r}\big (T(\vec {x}),\vec {\omega }_{r},\lambda \big ) \bigg ] + I_{bd}\end{aligned}$$

Because the beam dump is positioned far from the camera sharp field of view, one can consider that $I_{bd}$ **is negligible** ($I_{bd} \approx 0$). The radiative intensity emitted by any kind of opaque body in a direction $\vec {\omega _r}$ is the product of the black body radiative intensity (Eq. 1) and the directional emissivity ($\varepsilon '$) (Eq. 9):

8 $$\begin{aligned} I_e \big (T(\vec {x}),\vec {\omega }_r,\lambda \big )=\varepsilon '\big (T(\vec {x}),\vec {\omega }_r,\lambda \big )\, I_b\big (T(\vec {x}),\lambda \big ) \end{aligned}$$

The directional emissivity $\varepsilon '$ of an opaque surface is related to the directional-hemispherical reflectivity $\rho ^{\prime \cap }$^37,38^ by Kirchhof’s law, Eq. 10, whereas the relationship between $\rho ^{\prime \cap }$ and the BRDF ($\rho ''$) is given in Eq. 11:

9 $$\begin{aligned} \varepsilon ^\prime \big (T(\vec {x}),\vec {\omega }_r,\lambda \big )= & {} 1-\rho ^{\prime \, \cap }\big (T(\vec {x}),-\vec {\omega }_{r},\lambda \big ) \ \end{aligned}$$

10 $$\begin{aligned} \rho ^{\prime \cap }\big (T(\vec {x}),\vec {\omega }_i, \lambda \big )= & {} \int _{2\pi } \; \rho ''\big (T(\vec {x}),\vec {\omega }_{r} \, |\vec {\omega }_i,\lambda \big ) \; \left| \vec {n}\cdot \vec {\omega }_r \right| \; d\Omega (\vec {\omega }_r) \end{aligned}$$

where $\Omega$ is the solid angle (*sr*). As already presented in the literature^39,40^, $I_{r}$ is the reflected radiative intensity at $\vec {x}$ in the direction $\vec {\omega }_r$ and coming from incident directions $\vec {\omega }_i$:

11 $$\begin{aligned} I_{r}\big (T(\vec {x}),\vec {\omega }_{r} \, ,\lambda \big ) =\int _{2\pi } \rho ''\big (T(\vec {x}),\vec {\omega }_{r} \, |\vec {\omega }_{i},\lambda \big ) \; I_l\big (T(\vec {x}'),\vec {\omega }_{i},\lambda \big ) \,\left| \vec {n}\cdot \vec {\omega }_{i} \right| \, d\Omega (\vec {\omega }_i) \end{aligned}$$

where $I_l\big (T(\vec {x}'),\vec {\omega }_{i},\lambda \big )$ is a radiative intensity coming from the surroundings towards the sample and originates from $\vec {x}'$ due to multireflection and emission in the thermal scene. This quantity will be named $I_{scn} = I_l\big (T(\vec {x}'),\vec {\omega }_{i},\lambda \big )$.

Based on Eqs. 9–12 and considering that the camera is perpendicular to the sample ($\vec {\omega }_{r}= \perp$), Eq. 8 can be written as Eq. 13 ($I_{bs}=0$).

12 $$ I_l\big (T(\vec {x}),\perp ,\lambda \big )=  {} \tau _{bs} \bigg [ \big (1-\rho ^{\perp \cap })I_b\big (T(\vec {x}),\lambda \big ) +  \int _{2\pi } \rho ''\big (T(\vec {x}),\vec {\omega }_{r} \, |\vec {\omega }_{i},\lambda \big ) \; I_{scn}\big (T(\vec {x}'),\vec {\omega }_{i},\lambda \big ) \,\left| \vec {n}\cdot \vec {\omega }_{i} \right| \, d\Omega (\vec {\omega }_i) $$

From the general equation of the mixed emission reflection problem presented in Eq. 13, one can understand that the encountered problem is strongly linked to the directional aspect of the reflected luminous beam. We soon understand the complexity of the problem. Indeed, to access $\rho ^{\perp \cap }$ and $I_{r}$, one must determine all BRDFs. As this value depends on many parameters (temperature, surface condition, etc.), the measurement of the BRDF should be conducted as soon as those parameters vary, which is not suitable experimentally. Thus, one must find a way to ease the problem. An easy and versatile approach is to **consider only specular reflection**. This hypothesis is valid if the studied wavelengths are larger than the surface roughness^41^. This assumption is fortunately applicable in many cases because IR cameras work with long wavelengths.

#### Remark

To ensure that this condition is met, it is possible to magnify the difference between the wavelength of the camera and the surface roughness. To do so, one can use a camera with an MCT or SLS sensor that works at longer wavelengths ($8 - 14~\mu m$), or polish the surface. The literature shows that^42^ reflection can be considered mainly specular if the wavelength is five time higher than the root mean square surface roughness.

Thus, we introduce a Fresnel model^43^ described in Eq. 13, where $Fr(\perp )$ is the normal Fresnel reflectivity dependent on the incident angle; $\vec {R}(\vec {\omega }_{i},\vec {n}(\vec {x}))$ is the specular direction from the incident direction $\vec {\omega }_{i}$ and the surface normal $\vec {n}(\vec {x})$ and $\rho ''_F$ is the Fresnel BRDF.

13 $$\begin{aligned} \rho ''_F(\vec {x},\perp |\vec {\omega }_{i},\lambda ) = \frac{Fr(\vec {x},\perp ,\lambda ) \; \delta \big ( \perp -\vec {R}(\vec {\omega }_{i},\vec {n}(\vec {x})) \big ) }{\left| \vec {n}(\vec {x})\cdot \perp \right| } \end{aligned}$$

Considering the sample as an optical mirror with unknown reflectivity implies that for a given $\vec {\omega }_{r}$, there is only one $\vec {\omega }_{i}$ for which $\rho ''_F(\vec {x},\vec {\omega }_{r} \, |\vec {\omega }_{i},\lambda ) \ne 0$. In the specific case of a normal reflection ($\vec {\omega _r} = \perp$), the only incident direction ($\vec {\omega _i}$) for which $\rho ''_F(\perp \, |\vec {\omega }_{i},\vec {x},\lambda ) \ne 0$ is the normal incident direction ($\vec {\omega _r}\, = \, \vec {\omega _i} \,=\,\perp$). Thus, the specular BRDF expression (Eq. 13) is replaced in Eqs. 11 and 12 to lead to Eqs. 14 and 15, respectively. To simplify the notation, $\rho ''_F(\vec {x},\perp \, | \perp ,\lambda )$ will be denoted $\rho ^\perp _F (\vec {x},\lambda )$:

14 $$\begin{aligned} \rho ^{\perp \cap }\big (T(\vec {x}), \lambda \big )&= \rho ^\perp _F(T(\vec {x}),\lambda ) \end{aligned}$$

15 $$\begin{aligned} I_{r}\big (T(\vec {x}),\perp ,\lambda \big )&= \rho ^\perp _F (T(\vec {x}),\lambda ) \; I_{scn}^\perp \end{aligned}$$

Based on Eqs. 14 and 15, Eq. 12 can be written as:

16 $$\begin{aligned} I_l\big (T(\vec {x}),\perp ,\lambda \big )= & {} \tau _{bs} \bigg [ \bigg (1-\rho _{F}^\perp \big (T(\vec {x}) ,\lambda \big ) \bigg )\, I_b(T(\vec {x}),\lambda )~+~\rho _{F}^\perp \big (T(\vec {x}), \lambda \big ) I_{scn}^\perp \bigg ] \end{aligned}$$

As the camera integrates the radiative intensity field on its wavelength range [$\lambda _1$, $\lambda _2$] and a tiny opening angle $\Delta \Omega ^\perp$ around the sample’s surface normal, one can introduce the radiative flux measured by a camera pixel ${\dot{q}}^l$ as follows:

17 $$\begin{aligned} {\dot{q}}^l\big (T(\vec {x}), \perp \big ) = \int _{\Delta \Omega ^\perp } d\vec {\omega }_r \int _{\lambda _1}^{\lambda _2} \;\tau _{bs} \bigg [ \big (1-\rho _{F}^\perp (T(\vec {x}) ,\lambda ) \big )\, I_b (T(\vec {x}),\lambda )~+~\rho _{F}^\perp \big (T(\vec {x}), \lambda \big ) I_{scn}^\perp \bigg ]d\lambda \end{aligned}$$

### Illustration of the model for numerical cases

**Figure 9:**
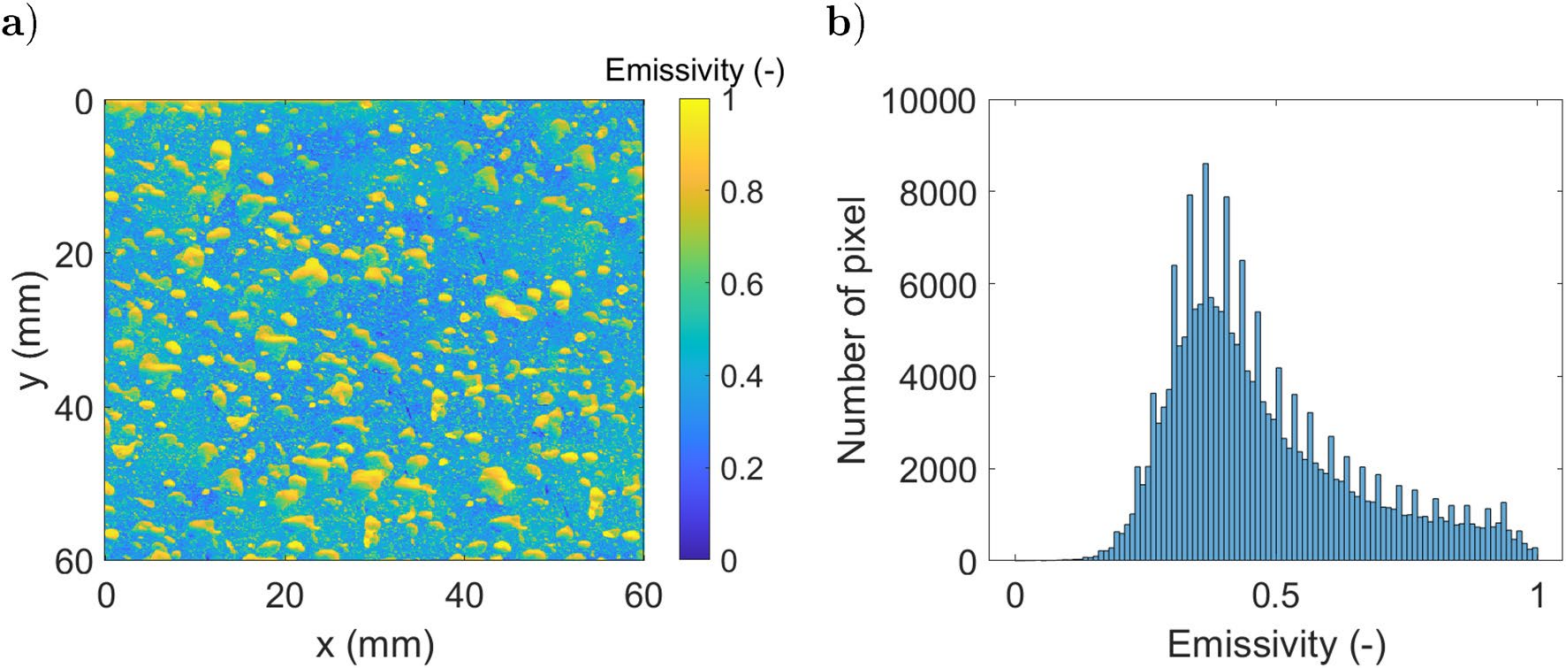
Presentation of the numerical study: (**a**) emissivity field, (**b**) histogram of emissivity.

Four numerical examples of thermography measurements are presented that implement the radiometric model described in Eq. 17. To represent cases of interest, a metal object at 300 *K* with a specular (optically smooth) surface is considered. The emissivity field is extracted from^44^. Figure 9.a shows the emissivity field, and Fig. 9.b shows the histogram of emissivity values.

The surrounding radiation coming from the scene is assumed to be given by Planck’s law at a uniform constant temperature, which can lead to three different cases.

1. The environment is at a lower temperature than the studied sample: $I_e(T(\vec {x}))~>~I_{scn}(T(\vec {x}'))$. To illustrate this situation, one can simulate a surrounding field at homogenous temperature (290 *K*), which carries a radiative intensity field $1.28~W.m^{-2}.sr^{-1}$.
2. The surroundings have a temperature similar to that of the sample: $I_e(T(\vec {x}))~\approx ~I_{scn}(T(\vec {x}'))$. To illustrate this situation, one can simulate a surrounding field at homogeneous temperature (300 *K*), which carries a radiative intensity field $1,87~W.m^{-2}.sr^{-1}$.
3. The sample is at a lower temperature than the surrounding sample: $I_e(T(\vec {x}))~ <~I_{scn}(T(\vec {x}'))$. To illustrate this situation, one can simulate a surrounding field at homogenous temperature (350 *K*), which carries a radiative intensity field $9,1~W.m^{-2}.sr^{-1}$.

**Figure 10:**
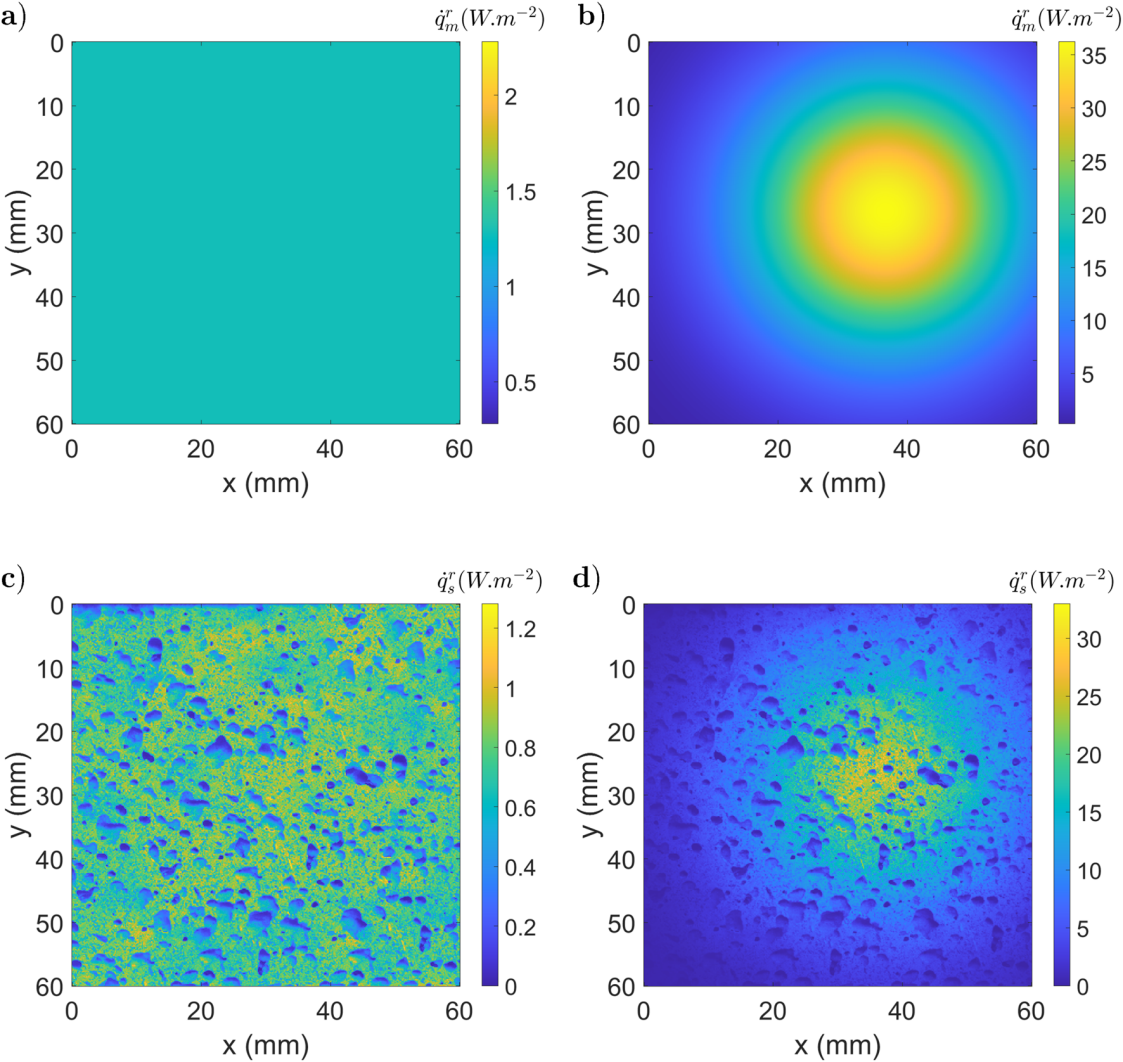
Surrounding radiative fluxes detected by the camera through a perfect mirror, (**a**) and (**b**), or through the heterogeneous sample, (**c**) and (**d**), in a homogeneous irradiation configuration (290 *K*), (**a**) and (**c**), or in a heterogeneous irradiation case, (**b**) and (**d**).

**Figure 11:**
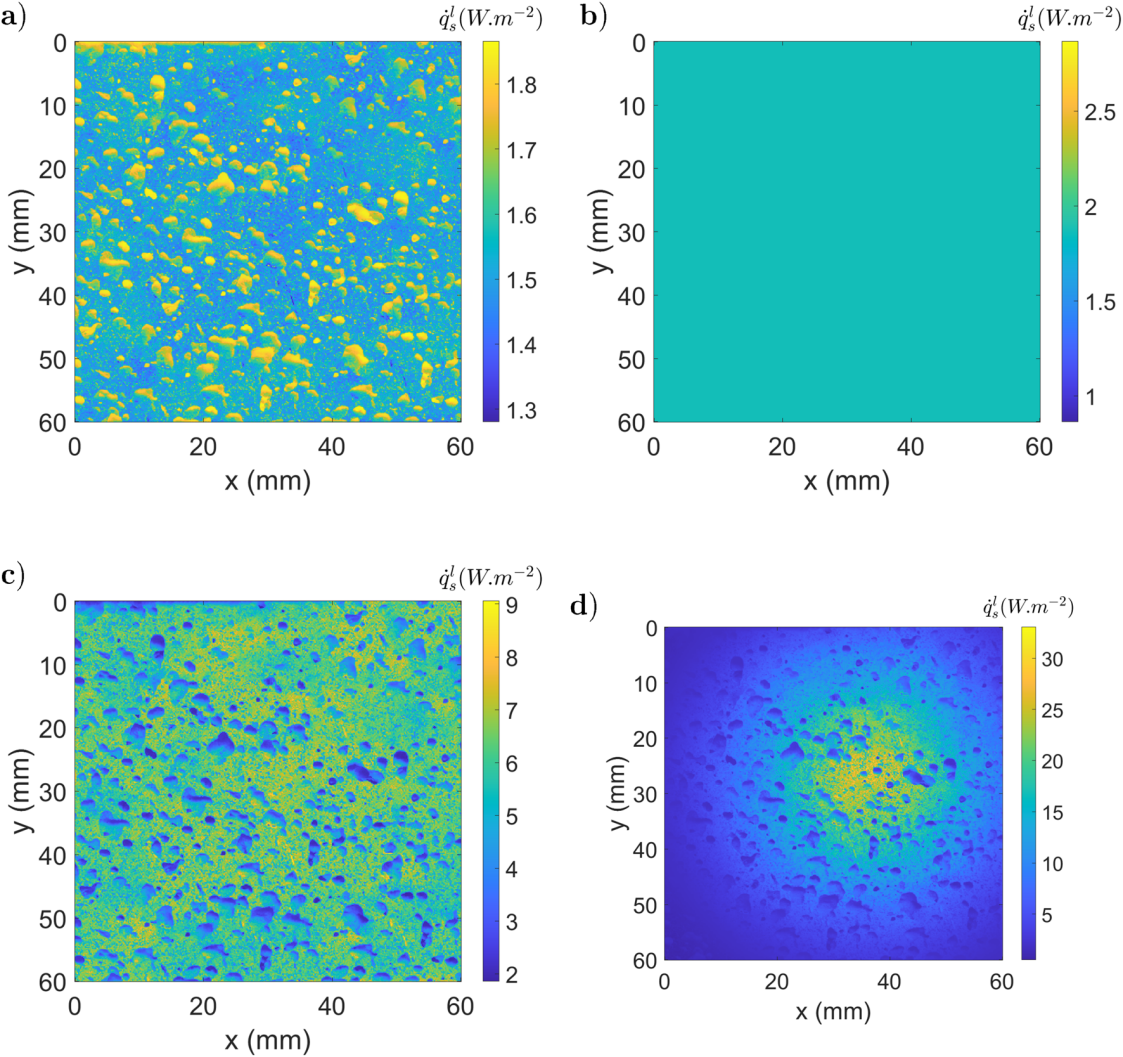
Radiative flux leaving (${\dot{q}}^l_s$) the sample (300 *K*) with different black body equivalent temperatures of the surrounding scene: (**a**) uniform at 290 *K*; (**b**) uniform at 300 *K*; (**c**) uniform at 350 *K*; (**d**) heterogeneous case.

A more realistic simulation of the surrounding environment considers multiple sources with a high variability of optical properties. This is the subject of the fourth simulated case where the heterogeneous surrounding field carries a radiative intensity field between 9, 1 and 14 $W.m^{-2}.sr^{-1}$. For each configuration, the radiative fluxes are obtained by integration of the radiative intensity between $\lambda _1 =2,5 ~\mu m$ and $\lambda _2 = 5~\mu m$ to simulate an IR camera sensor. The surrounding radiative fluxes detected by the camera through a perfect mirror (${\dot{q}}^r_m$: corresponding to the second term of Eq. 17) are presented in Fig. 10.a for the first configuration and Fig. 10.b for the fourth case. The surrounding radiative fluxes detected by the camera through the heterogeneous specular sample (${\dot{q}}^r_s$) are shown in Fig. 10.c for the first configuration and Fig. 10.d for the fourth case. Based on the emitted ($I_{e}$) and reflected ($I_{r}$) intensities, the radiative flux leaving the sample (${\dot{q}}^l_s~=~{\dot{q}}_l/\Delta \Omega ^\perp$) is computed for the four different (see Eq. 14) cases, as depicted in Fig. 11.

At this step, there is an unlikely conclusion. Indeed, if the surrounding irradiation is at the same temperature as the studied sample, the signal of the camera is uniform and correct on the whole image (case 2, Fig. 11). However, the measure is totally false because of the emissivity and the multiple reflections. This phenomenon can be explained with Eq. 17. Indeed, by considering $I_b \approx I_{scn}^\perp$, Eq. 17 can be written as Eq. 18.

18 $$\begin{aligned} {\dot{q}}^l_s\big (T(\vec {x}), \perp \big ) \approx \int _{\Delta \Omega ^\perp } d\vec {\omega }_r \int _{\lambda _1}^{\lambda _2} d\lambda \;\tau _{bs} \bigg [ I_b (T(\vec {x}),\lambda ) \bigg ] \end{aligned}$$

Thus, the reflected flux compensates for the emitted flux variation (due to emissivity), and the signal given by the camera is uniform. Although this case is numerical and the field obtained is perfectly homogeneous, it is representative of many experimental cases at room temperature with camera images being almost homogeneous despite heterogeneous emissivity fields. In this specific case, the measured field temperature seems almost constant and correct, whereas the measurement is biased by reflection.

On the other hand, in the other cases, one can clearly see that the radiative intensity leaving the sample is not sufficient to measure the temperature. For those reasons, we have developed a new method called Active Thermo-Reflectometry ( ATR), which is presented in the following.

### Method presentation

The ATR method searches to identify $T(\vec {x})$ from the radiometric model of Eq. 17. To achieve this goal, the proposed method includes 6 steps:

1. Determine a black body calibration relationship to link $\int d\lambda \, \tau _{bs}\, I_b\big (T(\vec {x}),\perp ,\lambda \big )$ to $T(\vec {x})$,
2. Acquire a raw image of the sample: ${\dot{q}}^l_s\big (T(\vec {x}),\perp \big )$,
3. Estimate the scene’s reflection on the sample: $\int d\lambda \,\tau _{bs}\,I_{scn}$,
4. Estimate the sample’s normal-normal reflectivity: $\rho _{F,s}^\perp$,
5. Compute the sample’s transmitted emittance: ${\dot{q}}_{s}^e$
6. Compute the sample’s transmitted black body emittance ${\dot{q}}_{s}^b$
7. Infer the sample’s true temperature with its emissivity and black body calibration.

The **first** and **second** steps have been described previously. Indeed, camera calibration is introduced in Sect. 2.3, and the acquisition of ${\dot{q}}_l$ is the raw image produced by the IR camera. In the **third step**, the radiative contribution ($I_{scn}$) of the surroundings is searched. To this aim, since the sample is assumed to be specular, we introduce ${\dot{q}}_{m,1}$, which corresponds to the pixel-based camera response during the closed chopper position when imaging the reference mirror.

19 $$\begin{aligned} {\dot{q}}_{m,1}\big (T(\vec {x}), \perp \big ) = \Delta \Omega ^\perp \int _{\lambda _1}^{\lambda _2} d\lambda \;\tau _{bs} \bigg [ \big (1-\rho _{F,m}^\perp (T(\vec {x}) ,\lambda ) \big )\, I_b (T(\vec {x}),\lambda )~+~\rho _{F,m}^\perp \big (T(\vec {x}), \lambda \big ) I_{scn}^\perp \bigg ] \end{aligned}$$

Where ${\lambda _1}$ and ${\lambda _2}$ are the bounds of the camera spectral window, $\Delta \Omega ^\perp$ corresponds to a pixel solid angle and $\rho _{F,m}^\perp$ is the mirror’s specular reflectivity. By considering the reference mirror with $\rho _{F,m}^\perp \approx 1$, its thermal emission may be neglected, and Eq. 19 can be simplified to:

20 $$\begin{aligned} {\dot{q}}_{m,1}\big (T(\vec {x}), \perp \big ) = \Delta \Omega ^\perp \int _{\lambda _1}^{\lambda _2} d\lambda \;\tau _{bs} \; I_{scn}^\perp \end{aligned}$$

To estimate ${\dot{q}}_{m,1}$, the reference mirror has to be placed in the exact same position and orientation as the sample. The **fourth step** is a key one. To determine the sample’s specular reflectivity, we introduce ${\dot{q}}_{s,1}$ and ${\dot{q}}_{s,2}$ that represent the pixel-based camera response with the chopper in closed or open positions, respectively. To express ${\dot{q}}_{s,2}$, we introduce the nearly collimated radiation $I_{BB}^\perp$ coming from the black body at a normal incident angle. Under these conditions, ${\dot{q}}_{s,1}$ and ${\dot{q}}_{s,2}$ are expressed in Eqs. 21 and 22.

21 $$\begin{aligned} {\dot{q}}_{s,1} \big (T(\vec {x}), \perp \big )= & {} \Delta \Omega ^\perp \int _{\lambda _1}^{\lambda _2} d\lambda \;\tau _{bs} \bigg [ \big (1-\rho _{F,s}^\perp \big (T(\vec {x}) ,\lambda ) \big )\, I_b (T(\vec {x}),\lambda )~+~ \nonumber \\&\rho _{F,s}^\perp \big (T(\vec {x}), \lambda \big ) I_{scn}^\perp \bigg ] \end{aligned}$$

22 $$\begin{aligned} {\dot{q}}_{s,2}\big (T(\vec {x}), \perp \big )= & {} \Delta \Omega ^\perp \int _{\lambda _1}^{\lambda _2} d\lambda \;\tau _{bs} \bigg [ \big (1-\rho _{F,s}^\perp (T(\vec {x}) ,\lambda ) \big )\, I_b (T(\vec {x}),\lambda )~+~ \nonumber \\&\rho _{F,s}^\perp \big (T(\vec {x}), \lambda \big ) \bigg ( I_{scn}^\perp + I_{BB}\bigg ) \bigg ] \end{aligned}$$

Then, the subtraction of the image taken without the reflection of the black body source on the sample (${\dot{q}}_{s,1}$) from the image with the source reflection (${\dot{q}}_{s,2}$) results in the elimination of the reflection of the surroundings and the sample’s thermal emission:

23 $$\begin{aligned} {\dot{q}}_{s,2}- {\dot{q}}_{s,1}= \Delta \Omega ^\perp \int _{\lambda _1}^{\lambda _2} d\lambda \;\tau _{bs}\;\rho _{F,s}^\perp \big (T(\vec {x}), \lambda \big )\, I_{BB} \end{aligned}$$

To evaluate $I_{BB}$, an additional measurement is performed on a reference mirror with a known reflectivity $\rho _{F,m}^\perp$ considered equal to unity:

24 $$\begin{aligned} {\dot{q}}_{m,2}- {\dot{q}}_{m,1}= \Delta \Omega ^\perp \int _{\lambda _1}^{\lambda _2} d\lambda \;\tau _{bs}\; I_{BB} \end{aligned}$$

The ratio of these differences (Eqs. 23–24) is used to evaluate the specular normal–normal reflectivity of the sample ($\rho _{F,s}^\perp$).

25 $$\begin{aligned} \frac{{\dot{q}}_{s,2}- {\dot{q}}_{s,1} }{{\dot{q}}_{m,2}- {\dot{q}}_{m,1}}= \frac{ \int _{\lambda _1}^{\lambda _2} d\lambda \; \rho _{F,s}^\perp \big (T(\vec {x}), \lambda \big )\, I_{BB} }{\int _{\lambda _1}^{\lambda _2} I_{BB}\,d\lambda } = {\tilde{\rho }}_{F,s}^\perp \big (T(\vec {x})\big ) \end{aligned}$$

This ratio corresponds to the averaged sample reflectivity weighted by the radiative intensity of the black body source and is denoted ${\tilde{\rho }}_{F,s}^\perp$.

To achieve the **fifth step**, the radiometric equation (Eq. 17) needs to be solved to obtain the black body radiative intensity of the sample. This requires the knowledge of ${\rho }_{F,s}^\perp$ (Eq. 25) and $I_{scn}^\perp$ (Eq. 20) and the assumption that $\rho _{F,s}^\perp$ is constant inside the wavelength range [$\lambda _1$, $\lambda _2$], leading to $\rho _{F,s}^\perp = {\tilde{\rho }}_{F,s}^\perp$. Thus, an expression of the sample’s emittance ${\dot{q}}_{s}^e$ transmitted to the camera in its spectral window may be written as:

26 $$\begin{aligned} {\dot{q}}_{s}^e = \Delta \Omega ^\perp \,\bigg (1-{\tilde{\rho }}_{F,s}^\perp \big (T(\vec {x}) \big ) \bigg ) \int _{\lambda _1}^{\lambda _2} d\lambda \;\tau _{bs}\, I_b (T(\vec {x}),\lambda ) \end{aligned}$$

and computed with Eq. 21, where the reflected surrounding radiative flux is removed using the reflectivity and Eq. 20:

27 $$\begin{aligned} {\dot{q}}_{s}^e = {\dot{q}}_{s,1} \big (T(\vec {x}), \perp \big ) -{\tilde{\rho }}_{F,s}^\perp \big (T(\vec {x})\big ) {\dot{q}}_{m,1}\big (T(\vec {x}), \perp \big ) \end{aligned}$$

The **sixth step** consists of dividing the sample transmitted emittance (${\dot{q}}_{s}^e$) by the constant sample emissivity ($1-{\tilde{\rho }}_{F,s}^\perp$) to compute the transmitted black body emittance (${\dot{q}}_{s}^b$) expressed in Eq. 28.

28 $$\begin{aligned} {\dot{q}}_{s}^b = \frac{{\dot{q}}_{s}^e}{1-{\tilde{\rho }}_{F,s}^\perp \big (T(\vec {x}) \big )} \end{aligned}$$

The **last step** makes use of the measured reflectivity and the previously recorded calibration relationship, Eq. 3, linking the sample’s true temperature to the sample’s black body equivalent emittance (${\dot{q}}_{s}^b$) transmitted to the camera (Eq. 2).

29 $$\begin{aligned} T(\vec {x}) = g\bigg [f\big ( {\dot{q}}_{s}^b \big ) \bigg ] \end{aligned}$$

### Numerical validation of ATR

**Figure 12:**
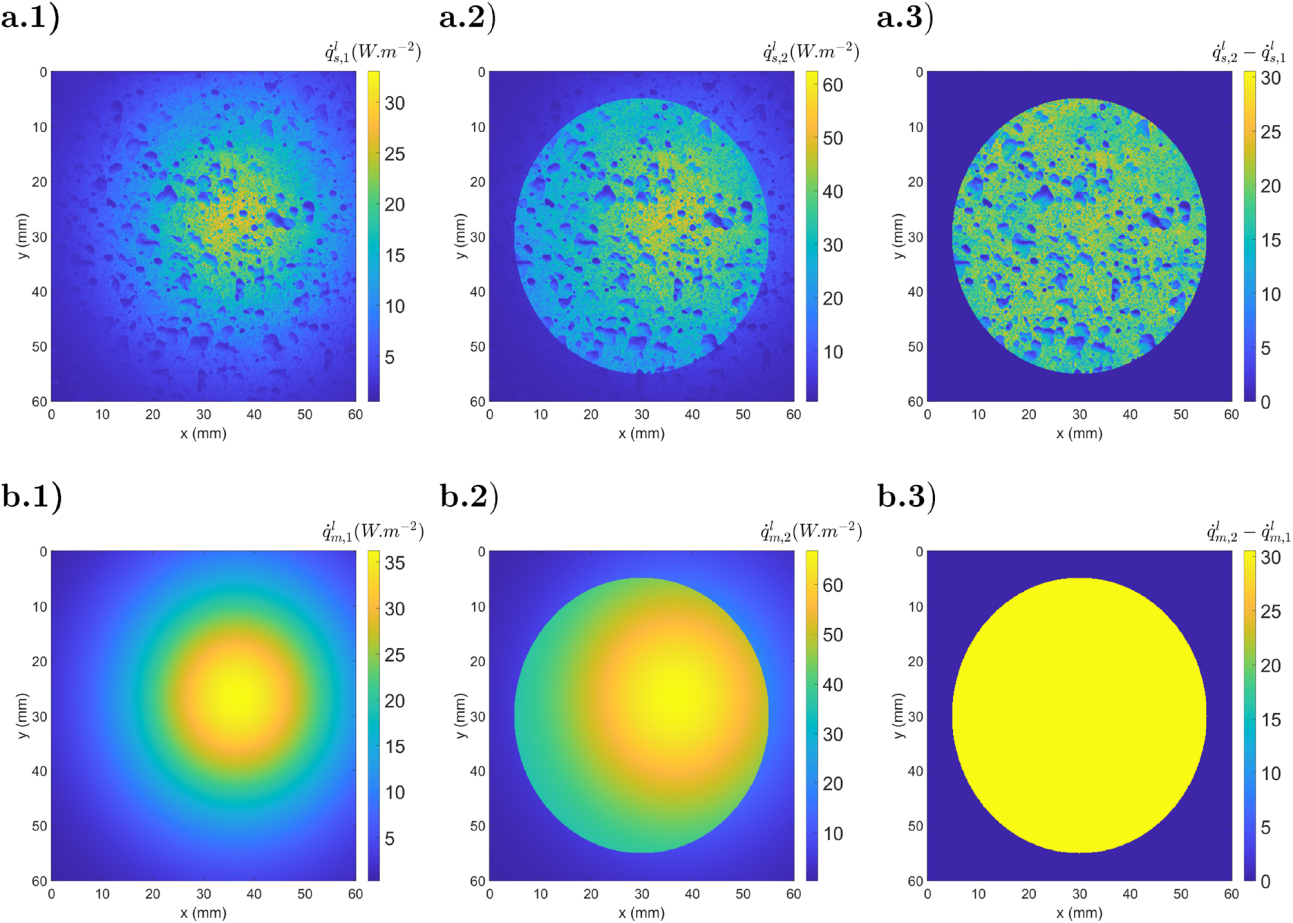
Simulated camera response (step 2 of ATR) for a heterogeneous sample (**a**) and a reference mirror (**b**): (a.1) radiative image from the sample ${\dot{q}}_{s,1}$; (a.2) radiative image from the illuminated sample ${\dot{q}}_{s,2}$; (a.3) difference between both images ${\dot{q}}_{s,2} - {\dot{q}}_{s,1}$; (b.1) radiative image from the mirror ${\dot{q}}_{m,1}$; (b.2) radiative image from the illuminated mirror ${\dot{q}}_{m,2}$; (a.3) difference between both images ${\dot{q}}_{m,2} - {\dot{q}}_{m,1}$.

The example studied in Sect. 3.2 will be used to numerically validate the ATR method. The illumination beam ($I_{BB}$) is simulated with normally collimated black body radiation at 400 *K*. Figure 12 shows the experimental part of the ATR method with the simulated infrared images of a heterogeneous specular sample and a reference mirror (steps 2, 3 and 4).

**Figure 13:**
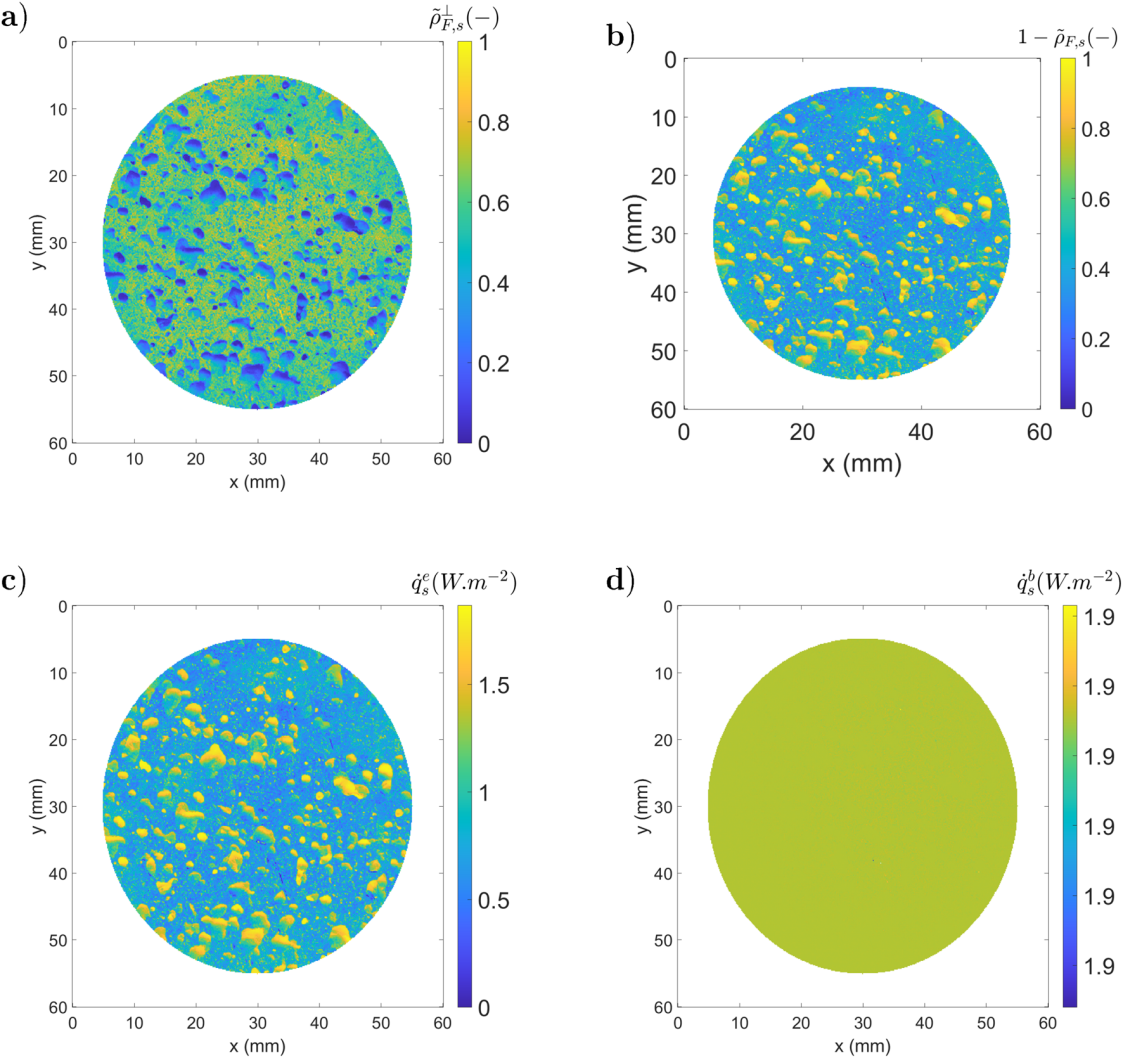
Illustration of the ATR method (**a**) ${\tilde{\rho }}_{F,s}^\perp$; (**b**) emissivity $(1-{\tilde{\rho }}_{F,s}^\perp )$; (**c**) ${\dot{q}}_{s}^e$; (**d**) ${\dot{q}}_{s}^b$.

Figure 13 corresponds to the method data processing. Fig. 13.a corresponds to the measurement of ${\tilde{\rho }}_{F,s}$ described in step 4, where the division of ${\dot{q}}_{s,2} - {\dot{q}}_{s,1}$ (Fig. 12.a.3) by ${\dot{q}}_{m,2} - {\dot{q}}_{m,1}$ (Fig. 12.b.3) is performed. Figure 13.b illustrates the measured emissivity maps obtained with Eq. 9. The determination of ${\dot{q}}_{s}^e$ (fifth step) is realized with ${\dot{q}}_{s,1}$ (Fig. 12.a.1), ${\dot{q}}_{m,1}$ (Fig. 12.b.1) and ${\tilde{\rho }}_{F,s}$ (Fig. 13.a) and shown in Fig. 13.c. Then, the black body equivalent emittance (${\dot{q}}_{s}^b$) was determined using ${\dot{q}}_{s}^e$ and the emissivity maps ($1-{\tilde{\rho }}_{F,s}^\perp$) described in Fig. 13.d.

As one can observe, the obtained emittance is constant along all specimens. Then, with the calibration curves, one can identify the temperature from ${\dot{q}}_{s}^b$ (Fig. 13.d). In this section, the steps of the ATR method are introduced in a theoretical study, whereas in the next section, they are applied to experimental images of actual samples.

## Results and discussion

### Stainless steel metallic sample

**Figure 14:**
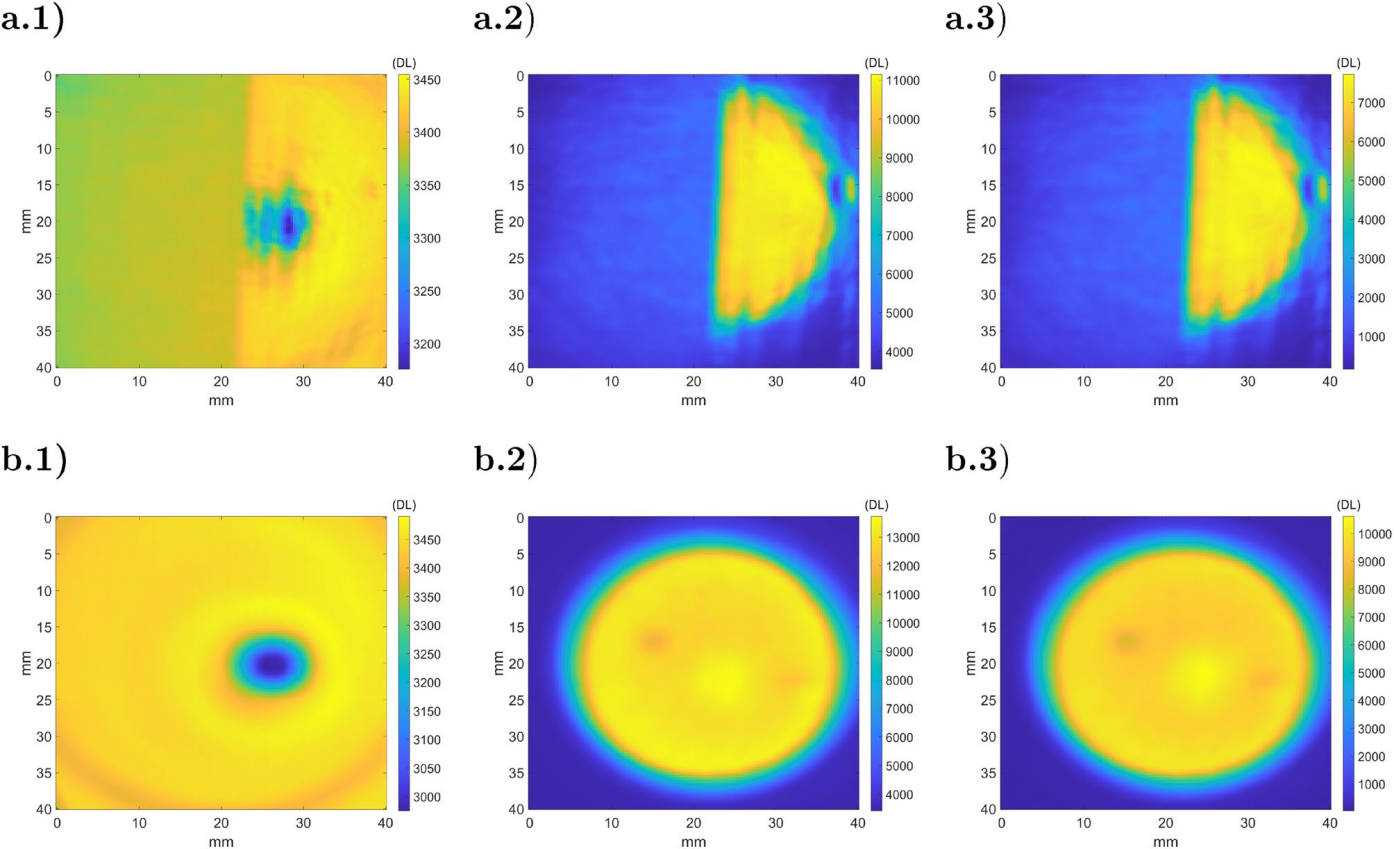
Camera measurements (step 2 of ATR) on the stainless steel metallic sample (**a**) and on the reference mirror (**b**): (a.1) ${\dot{q}}_{s,1}$; (a.2) ${\dot{q}}_{s,2}$; (a.3) ${\dot{q}}_{s,2} - {\dot{q}}_{s,1}$; (b.1) ${\dot{q}}_{m,1}$; (b.2) ${\dot{q}}_{m,2}$; (a.3) ${\dot{q}}_{m,2} - {\dot{q}}_{m,1}$.

This section shows the development of the ATR method based on the stainless steel metallic samples presented in Fig. 3.a. Figure 14 shows the results of step 2 in the ATR method with the recorded thermographic images for both the sample and reference mirror.

**Figure 15:**
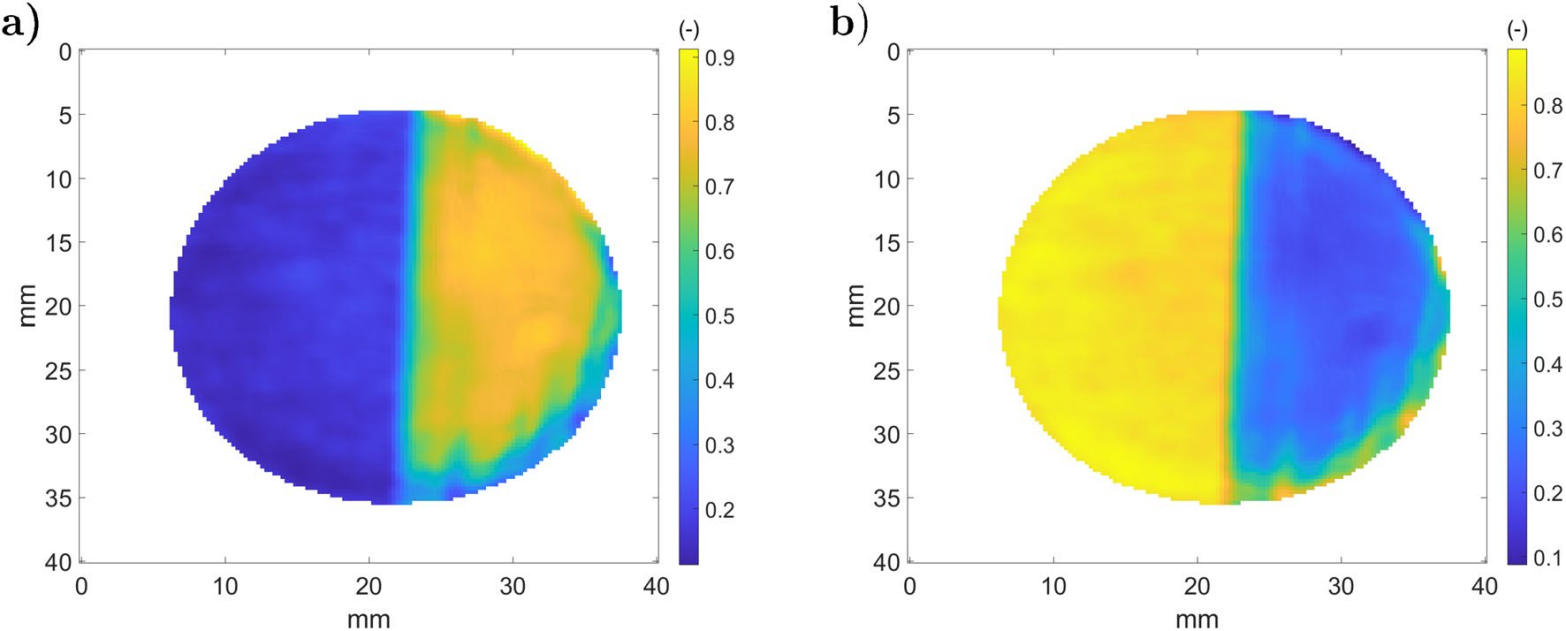
Measured sample radiative properties (step 4): (**a**) reflectivity ${\tilde{\rho }}_{F,s}^\perp$; (**b**) emissivity $(1-{\tilde{\rho }}_{F,s}^\perp )$.

Figure 14.a.1 shows the raw DL (proportional to ${\dot{q}}_{s,1}$) for the sample one. There are two parts in Fig. 14.a.3 corresponding to the rough (left) and laser polished (right) surfaces. Figure 14.b.1 shows ${\dot{q}}_{m,1}$), where a cold spot is visible due to the cooled camera narcissus^45^, which is also present in Fig. 14.a.1. Figure 14.a.3,b.3 show the transmitted reflections of the black body source for the sample and the mirror. Small heterogeneities of the source illumination are detected in Fig. 14.b.3 and are due to black body cavity heterogeneity. When the beam is reflected by the sample (Fig. 14.a.3), two surface treatments clearly appear. Based on the results of Fig. 14.a.3,b.3, the sample’s reflectivity and emissivity may be computed (step 4 of ATR), resulting in the radiative properties presented in Fig. 15.a,b.

Figure 15.a indicates that the polished part (on the right) of the sample is more reflective than the rough part. Based on the results of Fig. 15.a, the average total reflectivity and its standard deviation are computed for each part and gathered in Table 1. From Fig. 14.a.1, the polished part (on the right) of the sample appears warmer than the rough part, whereas the sample is isothermal.

**Table 1:** Measured effective reflectivity on stainless steel sample.

	Rough part	Polished part
${\tilde{\rho }}_{F,s}^\perp$	0.16	0.68
Standard deviation	0.02	0.12

**Figure 16:**
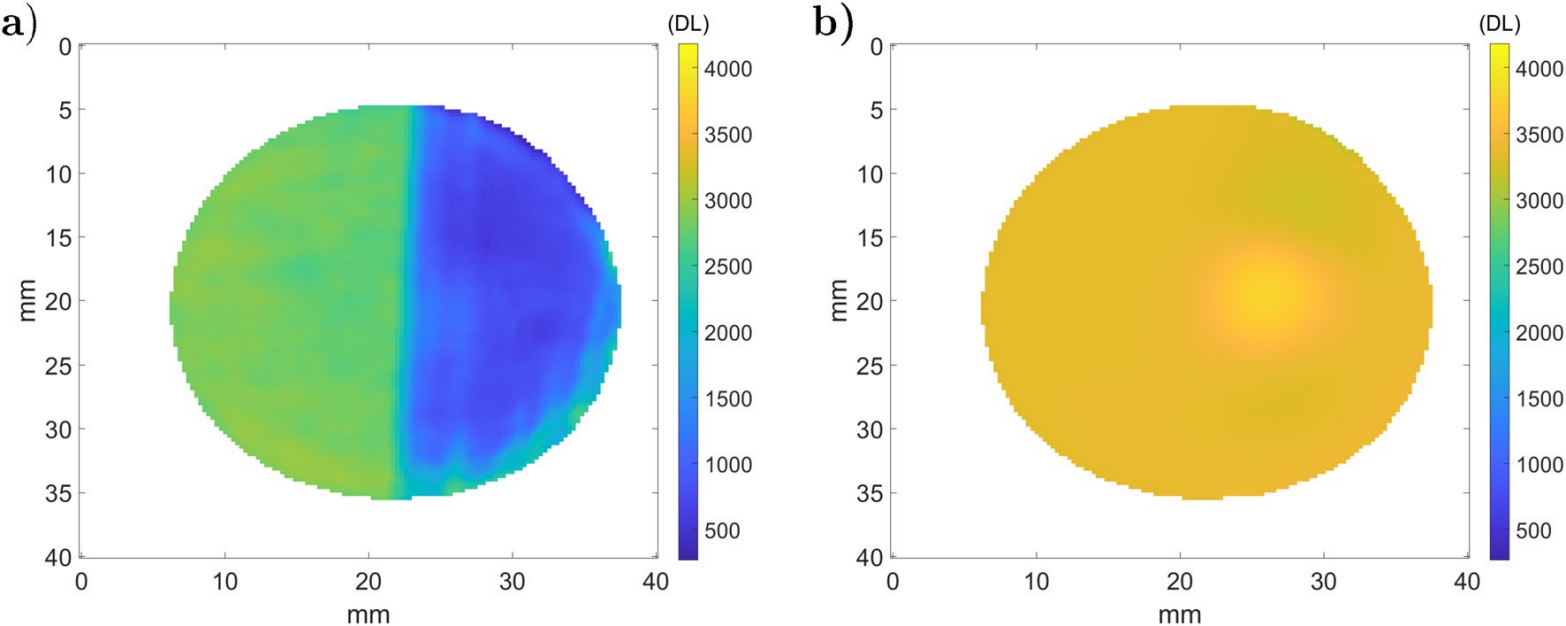
Measured stainless steel metallic sample emittance: (**a**) ${\dot{q}}_{s}^e$ (**b**) ${\dot{q}}_{s}^b$.

Figure 16.a presents the results of the ATR fifth step, ${\dot{q}}_{s}^e$, and the transmitted black body sample emittance in Fig. 16.b (sixth step). The rough part in Fig. 16.a corresponds to the region at 2500 DL, and the laser polished part is at 1000 DL. This is particularly interesting because it is opposed to the raw DL (Fig. 14.a.1), where reflection is dominant compared to emission.

As expected, ${\dot{q}}_{s}^b$ appears to be quasi-isothermal after correction by the sample’s emissivity (Eq. 29), and there is continuity between both rough and polished parts. Nevertheless, one can observe a brighter stain and some vertical lines on the polished part. The vertical lines are due to the laser path of the polishing process described in Fig. 3, which introduces errors in the reflectivity measurement that are amplified for highly reflective materials. Moreover, the brighter stain in the polished part of the sample is due to a failure with respect to camera narcissus attenuation.

**Figure 17:**
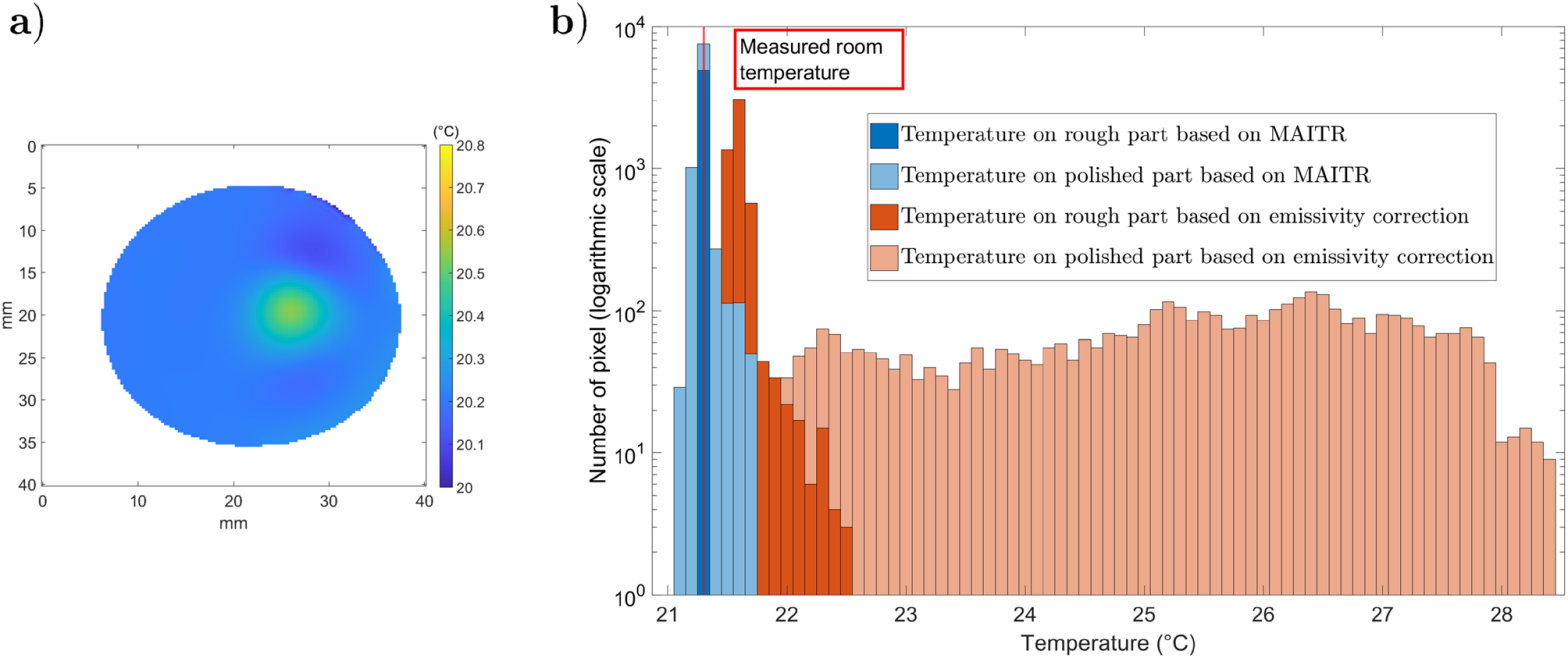
Sample temperature distribution ($^\circ$C): (**a**) temperature map; (**b**) histogram of temperatures for ATR and emissivity correction methods. The room temperature was measured as 21.3 °C.

Figure 17.a presents a map of the measured true temperatures computed with ATR, and Fig. 17.b shows a histogram of the measured temperature distribution obtained with ATR or emissivity correction only (${\dot{q}}_{s}^e/(1-{\tilde{\rho }}_{F,s}^\perp )$), not accounting for the surrounding reflection. With ATR, it is difficult to distinguish the temperature differences between the polished and rough parts. In addition, the error due to the narcissus has a negligible effect on the temperature evaluation. However, when applying the emissivity correction, even if the emissivity is known, the two parts are distinct. The measured temperature on the rough part fluctuates between 21.5 and 22.5 °C, whereas the measured temperature on the polished part is completely heterogeneous. The ATR method is found to be superior to the standard emissivity correction approach for specular materials at ambient temperature.

#### Remark

In this specific case, the assumption of a sample’s surface roughness being smaller than the wavelength is not satisfied for the rough part. Nevertheless, the results show that the reflections are mostly specular. If this had not been the case, one would have underestimated ${\tilde{\rho }}_{F,s}^\perp$ on this part. Thus, the corrected temperature would have been higher, and a gap between both parts would have appeared.

### Three-layer aluminium sample

**Figure 18:**
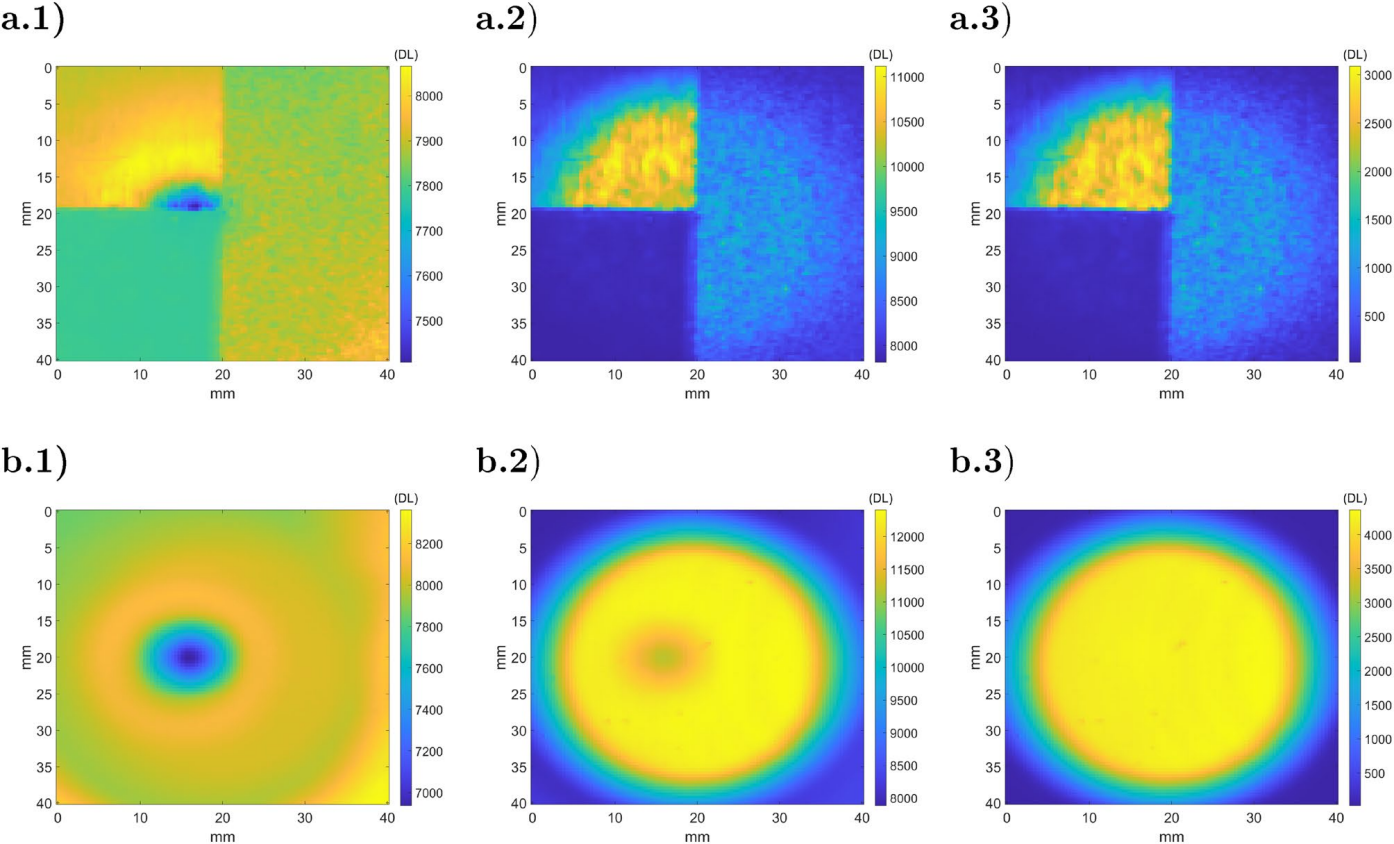
Camera measurements (step 2 of ATR) on the three-layer aluminum sample (**a**) and on the reference mirror (**b**): (a.1) ${\dot{q}}_{s,1}$; (a.2) ${\dot{q}}_{s,2}$; (a.3) ${\dot{q}}_{s,2} - {\dot{q}}_{s,1}$; (b.1) ${\dot{q}}_{m,1}$; (b.2) ${\dot{q}}_{m,2}$; (a.3) ${\dot{q}}_{m,2} - {\dot{q}}_{m,1}$.

**Figure 19:**
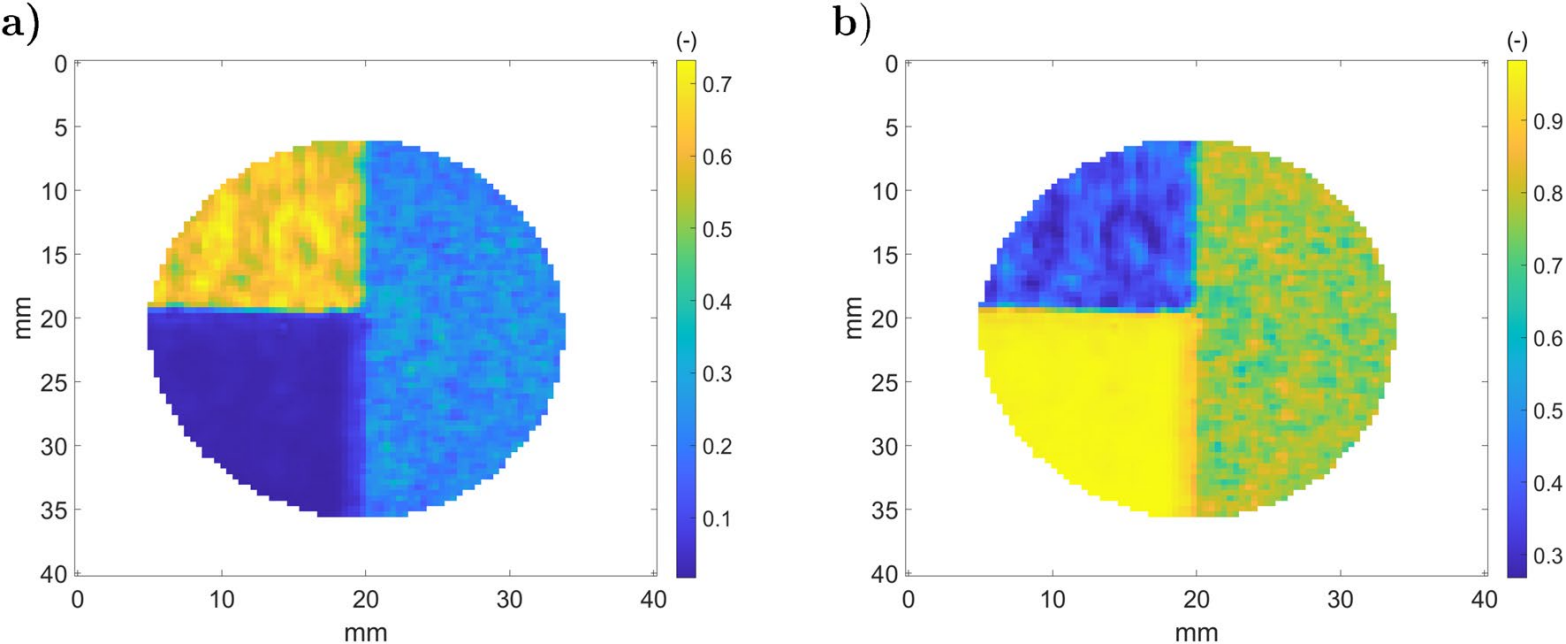
Measured sample radiative properties (step 4): (**a**) reflectivity ${\tilde{\rho }}_{F,s}^\perp$; (**b**) emissivity $(1-{\tilde{\rho }}_{F,s}^\perp )$.

This section shows the development of the ATR method on the three-layer aluminum sample presented in Fig. 3.b. Figure 18 shows the results of step 2 in the ATR method with the recorded thermographic images for both the sample and reference mirror. Figure 18.a.1,b.1 show images of the sample (${\dot{q}}_{s,1}$) and the mirror (${\dot{q}}_{m,1}$) with the chopper closed position whereas Fig. 18.a.2,b.2 correspond to the chopper open position (${\dot{q}}_{s,2}$ and ${\dot{q}}_{m,2}$). Figure 18.a.3,b.3 shows the difference for both the sample and the reference mirror. Based on this measurements, one can realise the fourth step of the ATR method and compute the normal reflectivity (Fig 19.a) and the normal emissivity (Fig 19.b). Figure 19.a indicates that the raw part is reflective ($\rho _{F,s}^\perp \approx 0.8$) and that the painted part has an emissivity value of approximately 0, 96, which is close to the value found in^25^. Surprisingly, the speckled area does not have such sharp reflectivity fields. This is due to the spatial resolution of the camera, which is lower than the size of the speckle pattern. Such problems are addressed in digital image correlation approaches^46^. Based on these results, an average effective reflectivity and its standard deviation for each part are computed and gathered in Table 2.

**Table 2:** Measured effective reflectivity on the aluminium three-part sample.

	Rough	Speckled	Painted
${\tilde{\rho }}_{F,s}^\perp$	0.62	0.23	0.04
Standard deviation	0.0546	0.0421	0.0227

**Figure 20:**
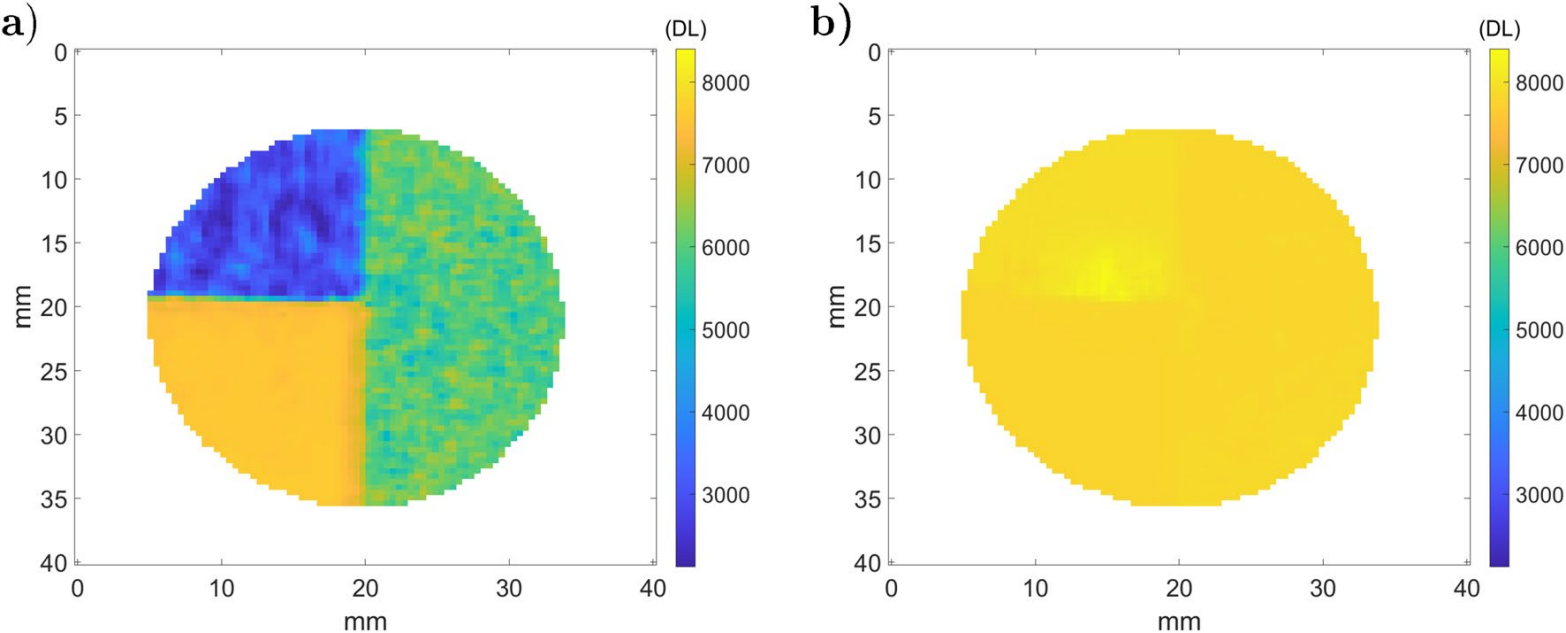
Measured emittance of the three-layer aluminum sample: (**a**) ${\dot{q}}_{s}^e$ (**b**) ${\dot{q}}_{s}^b$.

One can observe that the rough part has a higher total reflectivity than the speckled and uniformly painted parts. The standard deviation of the total reflectivity follows the same trend. Compared to the previous sample (stainless steel), the standard deviation is also lower. This reinforces the hypothesis that the method is sensitive to the surface flatness and noise measurement on highly reflective samples. From this measure, we can apply the fifth step of ATR and plot ${\dot{q}}_{s}^e$ and ${\dot{q}}_{s}^b$ in Fig. 20.a,b, respectively. Unlike the previous measurement, we do not use an average reflectivity on each sample part because of the high variability of the speckled surface. For this reason, the black body emittance is computed on a pixel basis and appears quasi-constant on the sample. Only a small area of the narcissus is still slightly visible.

**Figure 21:**
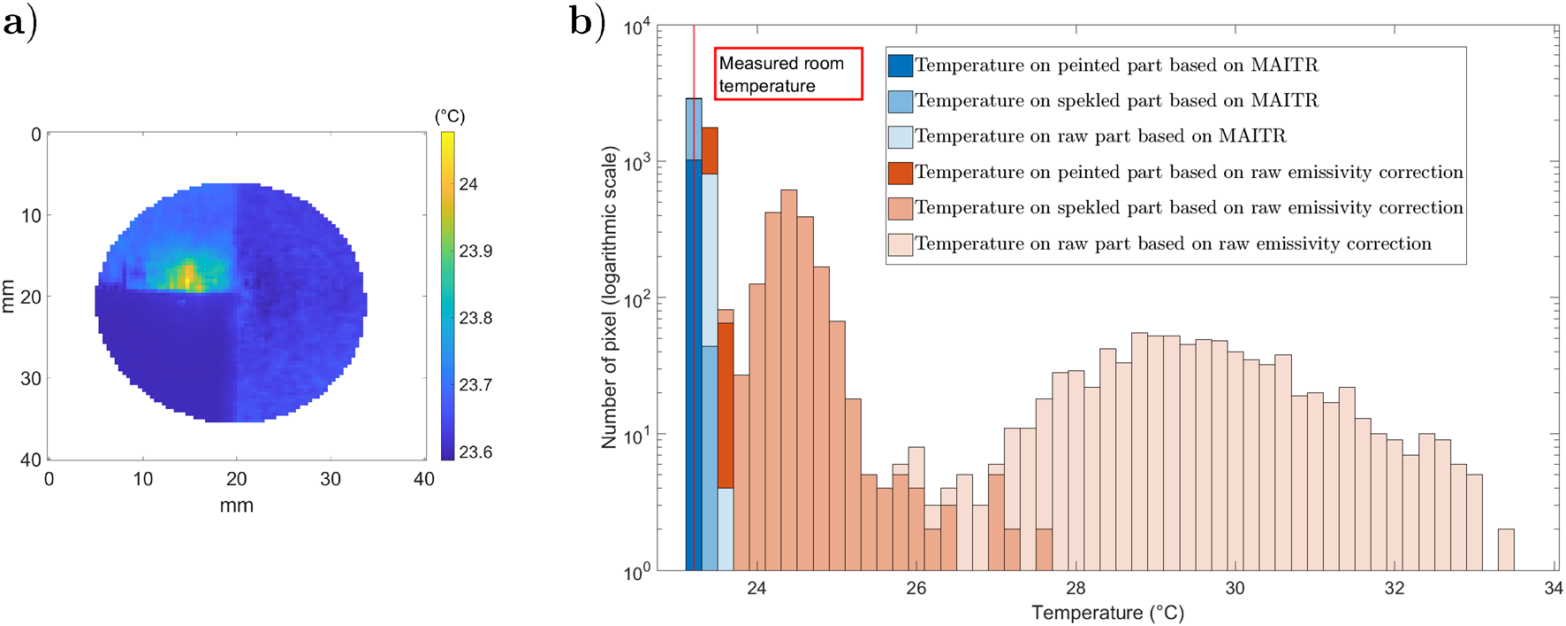
Sample temperature distribution ($^\circ$C): (**a**) temperature map; (**b**) histogram of temperatures for ATR and emissivity correction methods. The room temperature was measured as 23.2 $^\circ$C.

After converting the black body emittance with the last step of ATR, the temperature map is obtained and displayed in Fig. 21.a, and its histogram distribution is plotted in Fig. 21.b. The uniformity of the sample temperature is almost recovered by the ATR method, and the measured temperature corresponds to room temperature. Only the error due to the narcissus on the reflective raw part leads to a local overestimation of the sample’s true temperature. However, this error is negligible in the black-painted part of the sample since the reflection of the narcissus is negligible.

## Conclusion and perspectives

In this paper, we have developed a rapid and versatile method to characterize the normal emissivity on a specular opaque body for any type of IR camera over a wide range of wavelengths. The detailed ATR method allows simultaneous acquisition of the emissivity, an estimation of the reflections and temperature fields through an analytical radiation model and an experimental methodology.

We have seen that the raw images measured by IR cameras are not sufficient to easily obtain an absolute and remote measurement of the temperature. Indeed, the lack of local knowledge of the emissivity fields and the influence of reflections from the environment do not allow accurate radiometric measurement.

In this work, validation was carried out on various types of samples ranging from opaque materials such as black bodies to metallic materials such as polished sample and hybrid surfaces. For each case, the application of radiometric balances allowed us to highlight the contribution and interest of the ATR method for the absolute and contactless measurement of temperature fields by IR thermography. Moreover, the method has shown best results than emissivity correction only at ambient temperature because the reflection are not negligible.

The necessity of specular reflection as well as the normal alignment impose experimental precaution. Nevertheless, if this condition is respected, the method allows an absolute temperature measurement without any assumption. This is a main advantage comparing to other available method which are often based on correlation on nearby wavelength.

Further developments are underway to validate the method with respect to unsteady thermal processes in extreme conditions, as well as monochromatic multispectral approaches or roughness limitations.

## References

[1] Nicodemus FE (1965). Directional reflectance and emissivity of an opaque surface. Appl. Opt..

[2] Madding RP (1999). Emissivity measurement and temperature correction accuracy considerations. Thermosense XXI.

[3] Herne H (1953). The theoretical characteristics of bichromatic pyrometers. Br. J. Appl. Phys..

[4] Mekhrengin MV, Meshkovskii IK, Tashkinov VA, Guryev VI, Sukhinets AV, Smirnov DS (2019). Multispectral pyrometer for high temperature measurements inside combustion chamber of gas turbine engines. Measurement.

[5] Schmugge T, French A, Ritchie JC, Rango A, Pelgrum H (2002). Temperature and emissivity separation from multispectral thermal infrared observations. Remote Sens. Environ..

[6] Zhu C, Hobbs MJ, Willmott JR (2020). An accurate instrument for emissivity measurements by direct and indirect methods. Meas. Sci. Technol..

[7] Hanssen LM, Cagran CP, Prokhorov AV, Mekhontsev SN, Khromchenko VB (2007). Use of a high-temperature integrating sphere reflectometer for surface-temperature measurements. Int. J. Thermophys..

[8] Levick, A., & Edwards, G. A Fibre-optic based laser absorption radiation thermometry (LART) instrument for surface temperature measurement. In *Conference of Photoacoustic and Photothermal Phenomena* vol. 1, 438–441 (The Japan Society for Analytical Chemistry, 2002).

[9] Hernandez D (2005). A concept to determine the true temperature of opaque materials using a tricolor pyroreflectometer. Rev. Sci. Instrum..

[10] Monchau JP, Marchetti M, Ibos L, Dumoulin J, Feuillet V, Candau Y (2014). Infrared emissivity measurements of building and civil engineering materials: A new device for measuring emissivity. Int. J. Thermophys..

[11] Hernandez D, Sans JL, Pfänder M (2008). Pyroreflectometry to determine the true temperature and optical properties of surfaces. J. Sol. Energy Eng..

[12] Javaudin B, Gilblas R, Sentenac T, Le Maoult Y (2021). Experimental validation of the diffusion function model for accuracy-enhanced thermoreflectometry. Quant. InfraRed Thermogr. J..

[13] Sentenac T, Gilblas R, Hernandez D, Le Maoult Y (2012). Bi-color near infrared thermoreflectometry: A method for true temperature field measurement. Rev. Sci. Instrum..

[14] Jin H, Wang Y (2014). A fusion method for visible and infrared images based on contrast pyramid with teaching learning based optimization. Infrared Phys. Technol..

[15] Gao Y, Tian GY (2018). Emissivity correction using spectrum correlation of infrared and visible images. Sens. Actuators A Phys..

[16] Vellvehi M, Perpiñá X, Lauro GL, Perillo F, Jordá X (2011). Irradiance-based emissivity correction in infrared thermography for electronic applications. Rev. Sci. Instrum..

[17] Aumeunier M-H, Gerardin J, Talatizi C, Le Bohec M, Ben Yaala M, Marot L, Loarer T, Mitteau R, Gaspar J, Rigollet F, Courtois X, Houry M, Herrmann A, Faitsch M (2021). Infrared thermography in metallic environments of WEST and ASDEX Upgrade. Nucl. Mater. Energy.

[18] Talatizi C (2020). Inverse radiation problem with infrared images to monitor plasma-facing components temperature in metallic fusion devices. Fusion Eng. Des..

[19] Zeise, B., & Wagner, B. Temperature correction and reflection removal in thermal images using 3D temperature mapping. In *Proceedings of the 13th International Conference on Informatics in Control, Automation and Robotics* 158–165 (SCITEPRESS - Science and and Technology Publications, Lisbon, Portugal, 2016).

[20] Vollmer M, Henke S, Karstädt D (2004). Identification and suppression of thermal reflections in infrared thermal imaging. Inframation Proc..

[21] Meng D, Lin S, Azari H (2018). Reducing thermal reflections for infrared thermography applications on tunnel liners with reflective finishes. Transp. Res. Rec. J. Transp. Res. Board.

[22] Kirchner S, Narinsamy S, Sommier A, Romano M, Ryu M, Morikawa J, Leng J, Batsale JC, Pradére C (2018). Calibration procedure for attenuation coefficient measurements in highly opaque media using infrared focal plane array (irfpa) spectroscopy. Appl. Spectrosc..

[23] Pradere C, Ryu M, Sommier A, Romano M, Kusiak A, Battaglia JL, Batsale JC, Morikawa J (2017). Non-contact temperature field measurement of solids by infrared multispectral thermotransmittance. J. Appl. Phys..

[24] Husson, F., Valentin, M., Aouati, K., & Kling, R. Upscaling laser polishing of large 3D surfaces. In *High-Power Laser Materials Processing: Applications, Diagnostics, and Systems IX* (ed. Stefan K. & Stefan W. Heinemann) 4 (SPIE, San Francisco, United States, 2020).

[25] Jandrlić I, Rešković S (2015). Choosing the optimal coating for thermographic inspection. Holist Approach Environ..

[26] Aragon B, Johansen K, Parkes S, Malbeteau Y, Al-Mashharawi S, Al-Amoudi T, Andrade CF, Turner D, Lucieer A, McCabe MF (2020). A calibration procedure for field and UAV-based uncooled thermal infrared instruments. Sensors.

[27] Burggraaff O, Schmidt N, Zamorano J, Pauly K, Pascual S, Tapia C, Spyrakos E, Snik F (2019). Standardized spectral and radiometric calibration of consumer cameras. Opt. Express.

[28] Calik, R. C., Tunali, E., Ercan, B., & Oz, S. A study on calibration methods for infrared focal plane array cameras. In *VISIGRAPP*, 219–226, (2018).

[29] Lagüela S, González-Jorge H, Armesto J, Arias P (2011). Calibration and verification of thermographic cameras for geometric measurements. Infrared Phys. Technol..

[30] Machin G, Simpson R, Broussely M (2009). Calibration and validation of thermal imagers. Quant. InfraRed Thermogr. J..

[31] Malmivirta, T., Hamberg, J., Lagerspetz, E., Li, X., Peltonen, E., Flores, H., & Nurmi, P. Hot or Not? Robust and accurate continuous thermal imaging on FLIR cameras. In *International Conference on Pervasive Computing and Communications (PerCom)* 1–9 (IEEE, Kyoto, Japan, 2019).

[32] Marcotte F, Tremblay P, Farley V (2013). Infrared camera NUC and calibration: Comparison of advanced methods. Int. Soc. Opt. Photonics.

[33] Playá-Montmany N, Tattersall GJ (2021). Spot size, distance and emissivity errors in field applications of infrared thermography. Methods Ecol. Evol..

[34] Herrera D, Kannala J, Heikkila J (2012). Joint depth and color camera calibration with distortion correction. IEEE Trans. Pattern Anal. Mach. Intell..

[35] Souhar, Y. *Caractérisation thermique de matériaux anisotropes á hautes températures*. Doctoral dissertation, Institut National Polytechnique de Lorraine, (2011).

[36] Lane, B. M., & Whitenton, E. P. Calibration and measurement procedures for a high magnification thermal camera. Technical Report NIST IR 8098, National Institute of Standards and Technology, (2016). Issue: NIST IR 8098.

[37] Kirchhoff G (1860). On the relation between the radiating and absorbing powers of different bodies for light and heat. Lond. Edinb. Dublin Philos. Mag. J. Sci..

[38] Baltes HP, Wolf E (1976). On the validity of Kirchhoff’S law of heat radiation for a body in a nonequilibrium environment. Progress in optics.

[39] Caliot, C., Blanco, S., Coustet, C., El-Hafi, M., Eymet, V., Forest, V., Fournier, R., & Piaud, B. Combined conductive-radiative heat tranfert analysis in complex geometry using the Monte Carlo method. *hal-02096305ff*, 9, (2019).

[40] Michael F (2013). Modest, Radiative Heat Transfer.

[41] Bennett HE, Porteus JO (1961). Relation between surface roughness and specular reflectance at normal incidence. J. Opt. Soc. Am..

[42] Wen C-D, Mudawar I (2006). Modeling the effects of surface roughness on the emissivity of aluminum alloys. Int. J. Heat Mass Transf..

[43] Howell JR, Mengüç M, Daun K, Siegel R (2020). Thermal Radiation Heat Transfer.

[44] Yambangyang, P. Photo of Basalt stone background - - ID:59160725 - Royalty Free Image - Stocklib.

[45] Howard JW, Abel IR (1982). Narcissus: Reflections on retroreflections in thermal imaging systems. Appl. Opt..

[46] Crammond G, Boyd SW, Dulieu-Barton JM (2013). Speckle pattern quality assessment for digital image correlation. Opt. Lasers Eng..

